# Extracellular vesicles may provide an alternative detoxification pathway during skeletal muscle myoblast ageing

**DOI:** 10.1002/jex2.171

**Published:** 2024-08-21

**Authors:** María Fernández‐Rhodes, Emma Buchan, Stephanie D. Gagnon, Jiani Qian, Lee Gethings, Rebecca Lees, Ben Peacock, Andrew J. Capel, Neil R. W. Martin, Pola Goldberg Oppenheimer, Mark P. Lewis, Owen G. Davies

**Affiliations:** ^1^ School of Sport Exercise and Health Sciences, Loughborough University Loughborough UK; ^2^ School of Chemical Engineering, Advanced Nanomaterials Structures and Applications Laboratories, College of Engineering and Physical Sciences University of Birmingham Birmingham UK; ^3^ Waters Corporation Wilmslow UK; ^4^ School of Biological Sciences University of Manchester Manchester UK; ^5^ Medical School University of Surrey Surrey UK; ^6^ NanoFCM Co., LTD Nottingham Nottinghamshire UK

**Keywords:** ageing, extracellular vesicles, human primary cells, LC‐MS/MS, Raman spectroscopy, skeletal muscle

## Abstract

Skeletal muscle (SM) acts as a secretory organ, capable of releasing myokines and extracellular vesicles (SM‐EVs) that impact myogenesis and homeostasis. While age‐related changes have been previously reported in murine SM‐EVs, no study has comprehensively profiled SM‐EV in human models. To this end, we provide the first comprehensive comparison of SM‐EVs from young and old human primary skeletal muscle cells (HPMCs) to map changes associated with SM ageing. HPMCs, isolated from young (24 ± 1.7 years old) and older (69 ± 2.6 years old) participants, were immunomagnetically sorted based on the presence of the myogenic marker CD56 (N‐CAM) and cultured as pure (100% CD56^+^) or mixed populations (MP: 90% CD56^+^). SM‐EVs were isolated using an optimised protocol combining ultrafiltration and size exclusion chromatography (UF + SEC) and their biological content was extensively characterised using Raman spectroscopy (RS) and liquid chromatography mass spectrometry (LC‐MS). Minimal variations in basic EV parameters (particle number, size, protein markers) were observed between young and old populations. However, biochemical fingerprinting by RS highlighted increased protein (amide I), lipid (phospholipids and phosphatidylcholine) and hypoxanthine signatures for older SM‐EVs. Through LC‐MS, we identified 84 shared proteins with functions principally related to cell homeostasis, muscle maintenance and transcriptional regulation. Significantly, SM‐EVs from older participants were comparatively enriched in proteins involved in oxidative stress and DNA/RNA mutagenesis, such as E3 ubiquitin‐protein ligase TTC3 (TTC3), little elongation complex subunit 1 (ICE1) and Acetyl‐CoA carboxylase 1 (ACACA). These data suggest SM‐EVs could provide an alternative pathway for homeostasis and detoxification during SM ageing.

## 1 INTRODUCTION

Skeletal muscle (SM) accounts for about 30%–40% of total body mass and is implicated in structural support, locomotion, whole‐body glucose expenditure and energy metabolism. Age‐related changes in SM can reduce an individual's ability to perform everyday physical activities, increasing their risk of frailty and fractures while also aggravating underlying diseases such as osteopenia. Age‐related loss of muscle mass and strength (sarcopenia) affects around 10%–16% of people globally (Yuan & Larsson, 2023). Prevalence is predicted to increase to 20%–22% in the next 30 years, which will put an increased strain on healthcare systems worldwide (Ethgen et al., 2017; Larsson et al., 2019; Olshansky et al., 2008). As such, a better understanding of molecular variations occurring within SM as we age will be crucial if we are to effectively monitor and treat age‐related muscle loss.

Over the past two decades, the role of the SM secretome has become increasingly recognised in processes governing tissue synthesis and organisation, as well as in mediating the body's responses to physical activity, disease and metabolic stress. Myoblasts and myotubes within the SM release an array of growth factors and cytokines implicated in intercellular communication and SM maintenance that have been collectively termed ‘myokines’ (e.g., IGF1, IL‐6 or Irisin) (Gomarasca et al., 2020; Severinsen & Pedersen, 2020). In addition to free myokines, both myoblasts and myotubes release nanoparticles termed extracellular vesicles (EVs), which we shall henceforth refer to as SM‐EVs. EVs are nanosized particles delimited by a lipid bilayer and can be broadly separated into three subpopulations based on their size and biogenesis: exosomes (size range ∼30–150 nm, endosomal biogenesis), microvesicles (MVs: size range ∼100–1000 nm, plasma membrane blebbing) and apoptotic bodies (AB: size range ∼0.5–2 µm, apoptosis) (Raposo & Stoorvogel, 2013). However, due to their overlapping sizes and bio‐compositions, effectively differentiating between the three subgroups remains challenging, with the recommendation that they be collectively referred to as EVs (Théry et al., 2018; Welsh et al., 2024; Witwer et al., 2013). EVs have been widely observed to facilitate the transport of a diverse array of proteins, lipids, nucleic acids and metabolites between multiple cell types and tissues within the body (Raposo & Stahl, 2019). SM‐EV samples from murine C2C12 and human primary myoblasts have previously been associated with prominent SM markers, such as, myosin heavy chain (MyHC) and desmin (Forterre et al., 2014; Le Bihan et al., 2012). To date, studies have highlighted the roles of SM‐EVs in processes ranging from myogenic regulation and tissue remodelling (Aswad et al., 2016; Baci et al., 2020; Guescini et al., 2017; Le Bihan et al., 2012) to exercise adaptation (Conkright et al., 2022; Frühbeis et al., 2015; Garner et al., 2020; Trovato et al., 2019) and inter‐tissue and even inter‐organ communication (Jalabert et al., 2021; Lamichhane et al., 2015; Lara‐Castillo & Johnson, 2020; Maurel et al., 2017; Rome, 2022; Rome et al., 2019). Notably, SM‐EVs from human cellular models were shown to be enriched in proteins required for SM development, hypertrophy and regeneration, such as fibroblast growth factor 2 (FGF‐2) and insulin‐like factors (Choi et al., 2016; Le Bihan et al., 2012; Rome et al., 2019; Trovato et al., 2019). Furthermore, there is evidence to suggest that certain miRNAs are selectively enriched in SM‐EVs (myomiRNAs: miR‐1, miR‐133 or miR‐206), which function in the regulation of muscle homeostasis and function (Hanson et al., 2023; Matsuzaka et al., 2016; Mytidou et al., 2021). More recently SM‐EVs have been identified as candidate vehicles for the transport of myokines (Forterre et al., 2014; Maggio et al., 2023), with 50%–80% of identified myokines associated with plasma‐derived EV fractions (Safdar & Tarnopolsky, 2018).

EVs could provide an accessible, non‐invasive window to expose age‐related changes, with some preliminary evidence documented using in vitro and in vivo murine SM models (Alfonzo et al., 2022; Mytidou et al., 2021). For example, SM‐EV from C2C12 myoblasts have been associated with miR‐690, which functions in the regulation of myogenic transcription factors such as MyoD or Pax7 (Shao et al., 2022). Moreover, SM‐EVs obtained from myogenic and ‘aged’ serially expanded non‐myogenic C2C12 cultures revealed differences in their biochemical Raman fingerprints, with lipid and protein variations evident during early myogenic differentiation (Davies et al., 2021). In vivo, models have identified that SM‐EVs derived from the serum of aged mice (21–24 months old) showed altered transcriptomes and the presence of miRNAs (e.g., miRNA‐34a), which are associated with the modulation of muscle mass, strength and longevity (Fulzele et al., 2019; Sahu et al., 2021). However, translation of these findings in human SM systems is currently lacking (Wang et al., 2022). The only available human study to date highlighted that premature stress‐induced senescent human myoblasts were able to transfer pro‐inflammatory mRNAs to healthy cells, thereby providing the first evidence of SM‐EVs in human SM pathophysiology (Hettinger et al., 2021). Collectively, these examples highlight how variations in SM‐EV cargo and membrane biochemistry may provide a valuable and under‐unexplored window into age‐related changes in SM.

In this study, we aimed to provide the first comprehensive comparison of SM‐EVs isolated from male human primary skeletal muscle cells (HPMCs) isolated from donors aged between 21 and 25 years (young) and 65 and 72 years (old) to map changes associated with SM ageing. To achieve this aim, we utilised two SM in vitro models based on the presence of pure CD56^+^ (N‐CAM) cells or a previously optimised myogenic model combining 90% CD56^+^ cells with 10% CD56^−^ cells, which has previously been applied to study SM regeneration in vitro (Fleming et al., 2020; Rimington et al., 2021).

## 2 METHODS

### 2.1 Human primary muscle cells (HPMCs)

Human primary muscle cells (HPMCs) used in this study were derived from young (24 ± 1.7 years old) and older (69 ± 2.6 years old) males (ethics code: R17‐P177). All individuals underwent an overnight fast. Body fat percentage and fasting plasma glucose and serum insulin values were recorded for each participant (Table 1). HPMCs were isolated via explant culture from muscle biopsies of the vastus lateralis muscle, obtained using the Bergstrom technique (Tarnopolsky et al., 2011). HPMCs were grown on Matrigel (*354234, Corning, UK*) coated T75 flasks and cultured in growth medium (GM) [DMEM supplemented with 20% fetal bovine serum (FBS) and 1% penicillin/streptomycin (PS)]. Cells were grown to 80% confluence in GM and passaged using Accutase (ACC) (*25‐058‐CL, Corning, UK*). CD56^+^ cells were isolated using magnetic‐activated cell sorting (MACS) as previously described (Fleming et al., 2020; Martin et al., 2013; Sinanan et al., 2004). After separation, cells were pelleted and resuspended in 1 mL GM. The recovered CD56^+^ cellular fraction was seeded in Matrigel coated flasks, at a density of 4000 cells/cm^2^. Mixed populations (MP) consisting of 90% CD56^+^ cells and 10% CD56^−^ cells were also cultured in the manner described, using the same seeding parameters. This MP model benefited the quality of myogenic constructs and improved their regeneration capacity (Fleming et al., 2020). Upon reaching 100% confluence, GM was switched to myogenic differentiation medium (DM) [DMEM supplemented with 2% horse serum (HS) in EV‐depleted conditions (serum spun for 16 h at 120,000 ×*g*) and 1% of PS solution] (Fleming et al., 2020).

**TABLE 1:** Metabolic characteristics of young and old participants from which human primary muscle cells were derived.

Participant code	Group	Age	Body fat (%)	Glucose (mmol/L)	Insulin (µIU/mL)
**1**	Young	25	12.3	5.4	5.0
**2**		25	18.0	5.2	11.4
**3**		21	18.8	4.2	5.8
**4**		24	16.3	4.7	6.8
**5**		25	20.7	4.3	6.8
**1**	Old	72	22.8	5.7	9.8
**2**		65	25.9	5.9	11.7
**3**		68	23.5	4.6	3.4
**4**		69	22.4	5.9	5.5
**5**		70	30.5	4.7	6.4

### 2.2 Imaging and immunocytochemistry

Cultures were imaged using a Leica Microsystems Brightfield Microscope (*Leica, UK*) (*n* = 5 images), and myotube width was calculated using Image J 1.50a (*National Institutes of Health, USA*). For immunocytochemical analysis, cultures were washed with phosphate buffer saline (PBS) 1X and fixed in 4% formaldehyde solution *(F8775, Sigma–Aldrich, Merck, KGaA, Darmstadt, Germany)* for 30 min at room temperature (RT). Cells were permeabilised using 0.2% Triton‐X 100 *(X100, Sigma–Aldrich, UK)* in tris buffer solution (TBS) 1X. Primary anti‐myosin heavy chain (MyHC) antibody *(MAB4470, Novus Biologicals, Bio‐Techne Ltd, UK)* was added in TBS at 1:200 dilution to each plate and incubated overnight at RT. Cells were washed in 1X TBS and incubated with secondary antibody Alexa 488 goat anti‐rabbit *(A11008, Invitrogen, UK)* and 4,6‐diamidino‐2‐phenylindole (DAPI) (1:1000) *(10184322, FisherScientific, UK*) in TBS 1X for 1 h at RT. Fluorescence was detected and imaged using a Leica DM2500 microscope (*Leica, UK*), taking a minimum of six images per condition. Images displaying both DAPI (blue) and MyHC (green) staining were used for average myotube width calculations using ImageJ 1.50a (*National Institutes of Health, USA*). Images were submitted to MyoCount, an open‐source resource that operates in MATLAB, to calculate the fusion index (Murphy et al., 2019).

### 2.3 SM‐EV isolation: ultrafiltration (UF) and size‐exclusion chromatography (SEC)

Conditioned medium (CM) from HPMCs was collected every 24 h for a total period of 5 days after mature myoblasts could be observed (Figure S1). CM was spun at 2000 ×*g* for 20 min to eliminate cellular debris and stored at −80°C. CM was concentrated using a Vivaspin 20 (100 kDa) (*GE28‐9323‐63, Merck KGaA, Darmstadt, Germany*) ultrafiltration (UF) column (Fernández‐Rhodes et al., 2023). A concentrated sample was submitted to size‐exclusion chromatography (SEC) columns (*qEVoriginal/70 nm, IZON SCIENCE LTD, New Zealand*), collecting fractions 2 to 10. The resulting fractions were then re‐concentrated using the same UF protocol (Fernández‐Rhodes et al., 2023).

### 2.4 Zeta potential measurements

A Zetasizer Nano ZS (*Malvern Panalytical, UK*) was used for EV membrane zeta potential measurements, in order to understand particle integrity and potential surface modifications. Samples containing 50 µL SM‐EV suspension and 950 µL of DPBS were submitted to a DTS1070 folded capillary cell (*Malvern Panalytical, UK*). Capillary cells were washed with isopropanol and deionized water and then dried before applying the sample. A measurement time of 60 s and 50 mV at room temperature was applied in monomodal mode. Three repeats per sample/run condition were used to obtain zeta potential membrane values.

### 2.5 Bicinchoninic acid (BCA) protein assay

Pierce BCA Protein Assay Kit (*23227, ThermoFisher Scientifics, UK*) was applied, according to the manufacturer's instructions to estimate EV protein concentrations. 25 µL of each sample was loaded in a 96‐well plate, followed by 200 µL of BCA/copper complex solution. The absorbance was measured at 562 nm in a Thermo Scientific Varioskan Flash microplate reader *(ThermoFisher Scientific, UK)* using SkanIt Software 2.4.5 RE.

### 2.6 Western blot

The sample was prepared at a concentration of 1 µg/mL in sample buffer (*1610747, BioRad, UK*) and lysis buffer (LB) [0.5% Triton X‐100, EDTA 1X and protease inhibitors (*10085973, FisherScientific, UK*)]. Samples were boiled for 3 min at 98°C, and then separated via SDS‐PAGE using precast 4%–15% polyacrylamide gels (*4561083, BioRad, UK*). 5 µg of protein loaded per sample in all cases, with three replicates. Precision Plus Protein Dual Colour Standards were applied for the estimation of molecular weight (*1610374, BioRad, UK*). Proteins were transferred to polyvinylidene fluoride (PVDF) membranes (*11544996, FisherScientific, UK*) that were blocked in EveryBlot blocking buffer (*12010020, BioRad, UK*) and washed in TBS with 0.1% Tween20 (TBST) 1X (*Merck 655204‐100ML, FisherScientifics, UK*). Membranes were incubated with primary antibodies (Table S1) overnight at 4°C with light agitation. The following day, membranes were washed three times in TBST 1X and incubated with an appropriate secondary antibody for 1 h at RT (Table S1). Protein bands were detected through chemiluminescence using the ChemiDoc XRS+ system 3.2 (*1708265, BioRad, UK*) and Image Lab software 1.46 (*Life Science Research, BioRad, UK*). Image J 1.50a (*National Institutes of Health, USA*) was applied for WB band quantification.

### 2.7 ExoELISA Ultra

CD63 and CD81 levels were quantified using ExoELISA‐ULTRA kits (*EXEL‐ULTRA‐CD63‐1/ EXEL‐ULTRA‐CD81‐1, System Bioscience, UK*). Volumes of EV preparations corresponding to 5 µg were immobilised onto the wells of a 96‐microtitre plate and the assay was carried out according to the manufacturer's instructions.

### 2.8 Nano flow cytometry (NanoFCM)

A NanoAnalyzer U30 instrument (*NanoFCM Inc., Nottingham, UK*) equipped with dual 488/640 nm lasers was used to quantify particle concentration and fluorescence intensity of specific EV markers, CD9, CD63 and CD81. All samples were diluted to equal particle concentrations and stained with CD9‐FITC (1:100‐500) (*124809, BioLegend, UK*), CD63‐APC (1:100–500) (*ab233056, Abcam, UK*) or CD81‐APC (1:100‐500) (*104909, BioLegend, UK*). Fluorescent particles were detected using light by three bandpass filters: SSC‐488/10; FL1‐525/40; FL2‐670/30. Data were processed using nFCM Professional Suite v1.8 software, as previously described (Lees et al., 2022).

### 2.9 Raman spectroscopy

EVs were analysed and spectra were acquired using a Renishaw Invia Qontor confocal Raman microscope (*Renishaw PLC, UK*). This was equipped with a Leica DMLM microscope with a 785 nm excitation laser with an output power of 10 mV. 5 µL of EV sample was deposited onto an aluminium foil‐covered glass slide and allowed to air dry. Raman measurements were performed using a 50x objective, 1200 lines/mm diffraction grating in the spectral region of 750–1800 cm^−1^ centred at 1200 cm^−1^. A 10 × 10 map with 5 µm increment and 10 accumulations each of 1 s exposure was used to collect Raman spectra. WiRE 5.1 (*Renishaw PLC, UK*) was used to acquire all data and for the polynomial (5^th^ order) baseline subtraction and cosmic ray removal. Spectra were normalised using the standard normal variate (SNV) and *p*‐value calculations were generated and calculated using Python 3.7 (*Plone and Python, USA*). Student's *t*‐test was used to determine *p*‐values. and *p* < 0.05 was considered significant. Data were split into groups of young CD56^+^ v old CD56^+^ (Supplementary material), old mixed (MP) v young mixed (MP). Furthermore, we assessed Lipid‐to‐protein ratios by the coefficient of measured peaks at 1314 cm^−1^ and 1656 cm^−1^.

Principal component analysis (PCA) was carried out in Python using the Sklearn package. PCA was used in this work to analyse the sample measured Raman spectra and quantify the correlation between young and old SM EVs. This was achieved by transforming the spectral data to a new set of variables, the principal components (PCs), which are uncorrelated, and which are ordered so that the first three PCs retain most of the variation present in all the variables. In this study, the variables were formed from the intensity values across the Raman shifts to generate a correlation matrix containing a set of orthogonal PCs that capture the most relevant information thus facilitating data reduction and analysis. Raman spectra are structured as a matrix, with each row representing a sample, and each column a variable. Before that, PCA polynomial baseline subtraction was performed using the software Wire 5. The data were centred to remove any baseline shifts or offsets by subtracting the mean intensity value at each Raman shift from all data points. Next scaling was applied to ensure all variables had the same weight in the analysis. This involved dividing each variable by its standard deviation. Since PCA is based on the covariance structure of the data, it is calculated from the centred and scaled data. For a dataset with *p* Raman shift variables and *n* observations, the covariance between the two variables *i* and *j* was computed as follows.

$$Cov\left(X_{i},X_{j}\right)=\frac{1}{n-1}\sum_{k=1}^{n}\left(X_{ik}-{\bar{X}}_{i}\right)\left(X_{jk}-{\bar{X}}_{j}\right)$$

where *Cov*(*X_i_,X__j_*) is the covariance between variables *X_i_* and *X_j_*. *X_ik_* is the value of variable *X_i_* for the *k* ^th^ observation (*k* ^th^ is the variance‐maximizing direction orthogonal to the previous *k*). *X ®^i^* is the mean of variable *X_i_* across all observations. *n* is the number of observations.

The covariance matrix is a p × p matrix, where each element (*i*,*j*) represents the covariance between variables *X__i_* and *X__j_*. The diagonal elements of the matrix contain the variances of each variable. The resulting covariance matrix is symmetric, where the element at row *i* and row *j* is equal to the element at row *j* and row *i*. It describes how each Raman shift variable is related to every other Raman shift variable in the dataset. This yields eigenvalues and eigenvectors with the eigenvectors representing the PCS and the eigenvalues indicating the importance or variance explained by each PC. Selection was based on a combination of eigenvalues and scree plot with PCs of relatively large eigenvalues selected for analysis to provide quantitative prediction/classification of datasets.

### 2.10 Liquid chromatography with mass spectrometry (LC‐MS) analysis

Ice‐cold acetone (400 µL) was added to EV samples suspended in PBS after isolation and then stored at −80°C for 1 h. Following incubation, samples were subject to BCA analysis to normalise for protein concentration and the preparation was standardised at 1 µg/mL. For protein digestion, 0.2% RapiGest (*715000122, Waters, UK*) was reconstituted in 50 mM of Ammonium Bicarbonate (pH 7.8) to make a stock solution. Samples were centrifuged at 14,000 ×*g* for 10 min and the supernatant was removed. Tubes were inverted to air dry the resulting pellet. 50 µL of RapiGest stock solution was applied to resuspend the dried protein pellets. Samples were heated at 80°C in a dry block for 45 min and centrifuged at 14,000 ×*g* for 10 min, with the supernatant recovered. Dithiothreitol (DTT) was added to the supernatant at a final concentration of 5 mM. Samples were heated for protein denaturation at 65°C for 20 min and left to cool at RT. Iodoacetamide was added to a final concentration of 15 mM and samples were left in the dark at room temperature for 30 min. Proteins were digested using 1 µg of Trypsin (*Pierce Trypsin Protease, MS Grade, 90057, ThermoFisher Scientific UK*) per 100 µg of protein overnight at 37°C. Finally, samples were acidified with formic acid to a final concentration of 0.5% v/v, incubated at 37°C for 25 min and centrifuged at 21,000 ×*g* for 20 min. The resulting supernatant was stored at −80°C.

### 2.11 LC‐MS configuration

One‐dimensional nanoscale LC separation of tryptic peptides was performed using an ACQUITY M Class system (*Waters Corporation, Milford, MA, USA*), equipped with a Symmetry C18 5 µm, 2 cm × 180 µm pre‐column and a High Strength Silica (HSS) T3 C18 1.7 µm, 15 cm × 75 µm analytical reversed‐phase (RP) column (*Waters Corporation, Milford, MA, USA*). Samples were transferred with aqueous 0.1% (v/v) formic acid to the pre‐column at a flow rate of 15 µL/min for 2 min. Mobile phase A was water containing 0.1% (v/v) formic acid, whilst mobile phase B was acetonitrile containing 0.1% (v/v) formic acid. Peptides were eluted from the pre‐column and separated with a gradient of 3%–40% mobile phase B over 60 min at a flow rate of 400 nL/min. The analytical column temperature was maintained at 35°C. Lock mass solution was delivered by the auxiliary pump of the LC system at 1 µL/min to the reference sprayer of the source of the mass spectrometer.

Mass spectrometric analysis was performed using a SELECT SERIES Cyclic Ion Mobility mass spectrometer (*Waters Corporation, Wilmslow, United Kingdom*). For all measurements, the mass spectrometer was operated in v‐mode with a nominal resolution of 35,000 full width at half maximum (FWHM). All analyses were performed in positive mode Electrospray Ionization (ESI). The ion source block temperature and capillary voltage were set to 100°C and 3.2 kV, respectively. The time‐of‐flight analyser of the mass spectrometer was externally calibrated with a NaCsI mixture from m/z 50 to 1990. The data were post‐acquisition lock mass‐corrected using the doubly charged monoisotopic ion of [Glu1]‐Fibrinopeptide B. The reference sprayer was sampled with a frequency of 60 s. Accurate mass LC‐MS data were collected in a randomised order using the ion mobility‐enabled, data‐independent acquisition mode (HDMSE) (Distler et al., 2014; Rodriguez‐Suarez et al., 2013). The spectral acquisition time in each mode was 0.5 s with a 0.02 s interscan delay. In low energy MS mode, data were collected at the constant trap and transfer collision energy of 6 eV (per unit charge). In the elevated energy mode, the trap collision energy was ramped from 19 to 45 eV (per unit charge) in 0.5 s. One cycle of low and elevated energy data was acquired every 1 s.

### 2.12 Data processing and database searching

Progenesis QI for Proteomics version 4.2 (*Nonlinear Dynamics, Newcastle upon Tyne, UK*) was used to process all the data acquired. Protein identifications were obtained by the reviewed entries of a Homo Sapiens UniProt database (20,405 reviewed entries, release 2022_12). To detect and monitor protein and peptide identification error rates (1% FDR), decoy database strategies were utilised as previously described (Li et al., 2009). Peptide and fragment ion tolerances were determined automatically, one missed cleavage site was allowed, as well as fixed modification carbamidomethylation of cysteine. Variable modifications were also specified, which included the oxidation of methionine and deamidation of asparagine and/or glutamine. From the abundance data obtained by Progenesis, linear regressions were plotted using Origin Lab 2020. Protein classification was performed using the Gene Ontology (GO) knowledgebase (http://geneontology.org/) and PANTHER (Protein ANalysis THrough Evolutionary Relationships) (http://pantherdb.org/). Protein lists were submitted to FunRich (http://www.funrich.org/), a software tool to describe their functional enrichment and the comparison of the proteome against Vesiclepedia (http://microvesicles.org/). Then, they were input to the web resource Metaboanalyst (http://www.metaboanalyst.ca) for a complete data and differential expression analysis, obtaining PCAs and heatmaps. This was achieved by selecting Log transformation and Pareto scaling data normalisation. STRING (https://www.string‐db.org) was used to obtain biological networks that SM‐EV proteomes may be involved in, using medium confidence (0.400).

### 2.13 Statistical analysis

Plots and graphs were generated using Origin Lab 2020 9.7.0.188 (*OriginLab Corporation, USA*) or GraphPad Prism 6 (GraphPad Software, San Diego, USA), with values presented as mean ± standard deviations (SDs). A 95% confidence interval (CI) was used in all functions. Pearson's correlation‐r was used for correlations within samples. Student's *t*‐tests (two‐tailed) and analysis of variance (ANOVA) with Bonferroni post‐hoc were performed using GraphPad Prism 6 (*GraphPad Software, San Diego, USA*). Differences were considered statistically significant at *p < 0.05, **p < 0.01 or ***p < 0.001. Metaboanalyst analyses were performed in the section Statistical Analysis [one factor], normalising the data with Log transformation and Pareto scaling. We selected the top 25 proteins for the Heatmaps.

## 3 RESULTS

### 3.1 Identification of myogenic characteristics in HPMCs from young and old individuals

**FIGURE 1:**
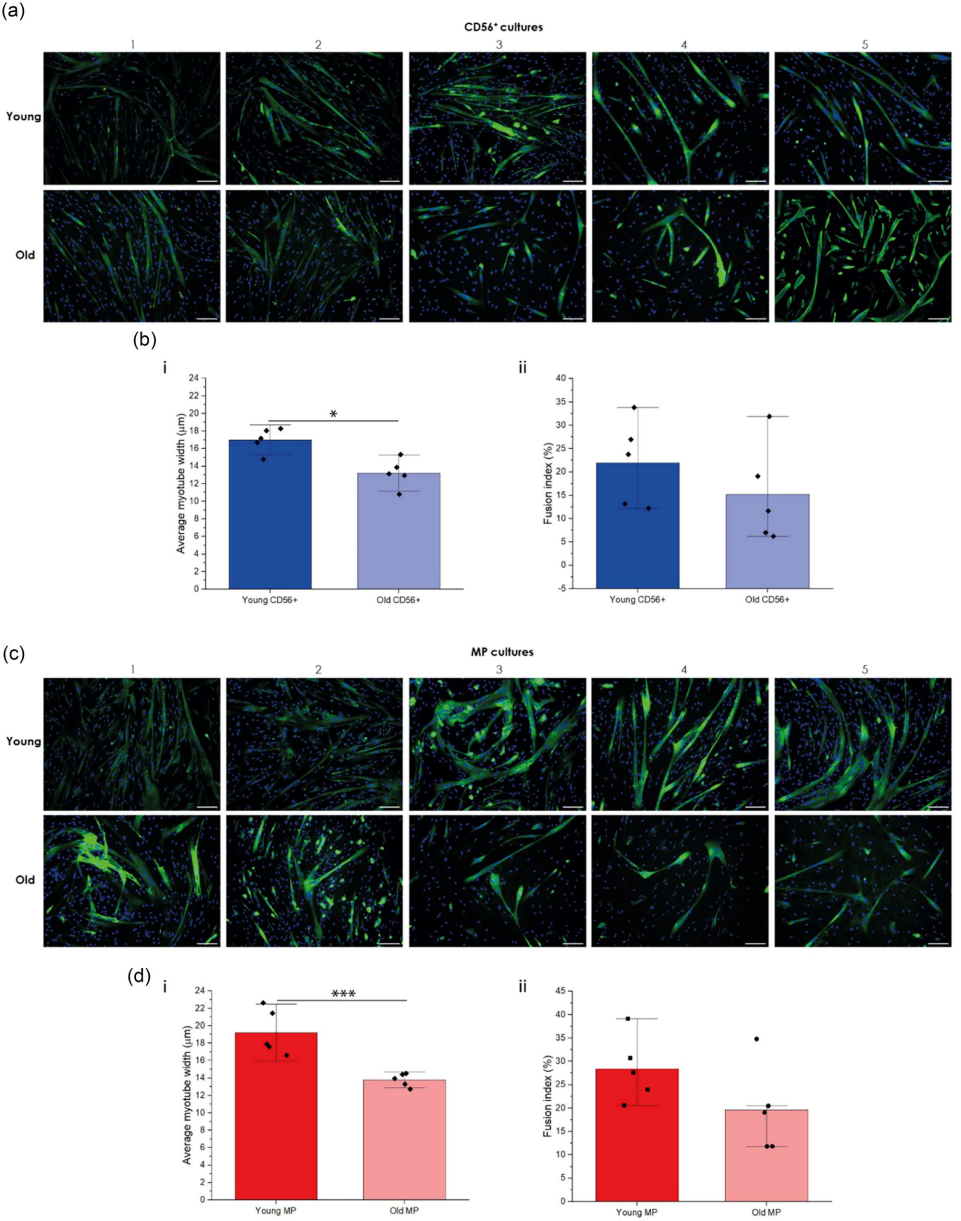
Myogenic identification of young and old HPMC cultures. (a) Images panel representing fluorescence microscopy images for CD56^+^ cultures (*n* = 5, young and old HPMCs) (green, MyHC and blue, DAPI). (b) Myogenic characteristics. (i) Average myotube width measurements for CD56^+^ cultures and (ii) fusion index measurements for CD56^+^ cultures. Fusion indices for young and old cohorts were calculated using MyoCount (*n* = 6 images) (MATLAB). (c) Images panel representing fluorescence microscopy images for MP cultures (*n* = 5, young and old HPMCs) (green, MyHC and blue, DAPI). (d) Average myotube width measurements for MP cultures. (ii) Fusion index measurements for MP cultures fusion indices for young and old cohorts were calculated using MyoCount (*n* = 6 images) (MATLAB). Scale bars = 50 µm.

HPMCs from both young and old participants showed visual evidence of myogenic differentiation after 5 days in culture, irrespective of their baseline characteristics (Figure 1). CD56^+^ cells from young and old cultures formed myotubes, as evidenced by MyHC fluorescence (Figure 1a). Average myotube width reflected significant differences between young (16.99 ± 1.39 µm) and older (13.21 ± 1.65 µm) CD56^+^ HPMCs (*p* < 0.05) (Figure 1b(i)). Fusion index values were 20.94 ± 3.04% and 15.17 ± 4.43% for young CD56^+^ and older CD56^+^ HPMCs, respectively (*p* > 0.05) (Figure 1b(ii)). Young MP samples displayed similar profiles, with significant differences observed in myotube width (19.22 ± 2.64 µm for young MP vs. 13.78 ± 0.76 µm for older MP, *p* < 0.001)) (Figure 1d(i)). Fusion indices were lower in older MP cultures (28.40 ± 4.45% for young vs. 19.60 ± 6.74% for old), although no significant differences were observed (Figure 1d(ii)). In these results, we showed a trend towards reduced myotube fusion in the older HMPC samples, which was also true for CD56^+^ models (Figure S2).

### 3.2 Comparison of EV profiles

**FIGURE 2:**
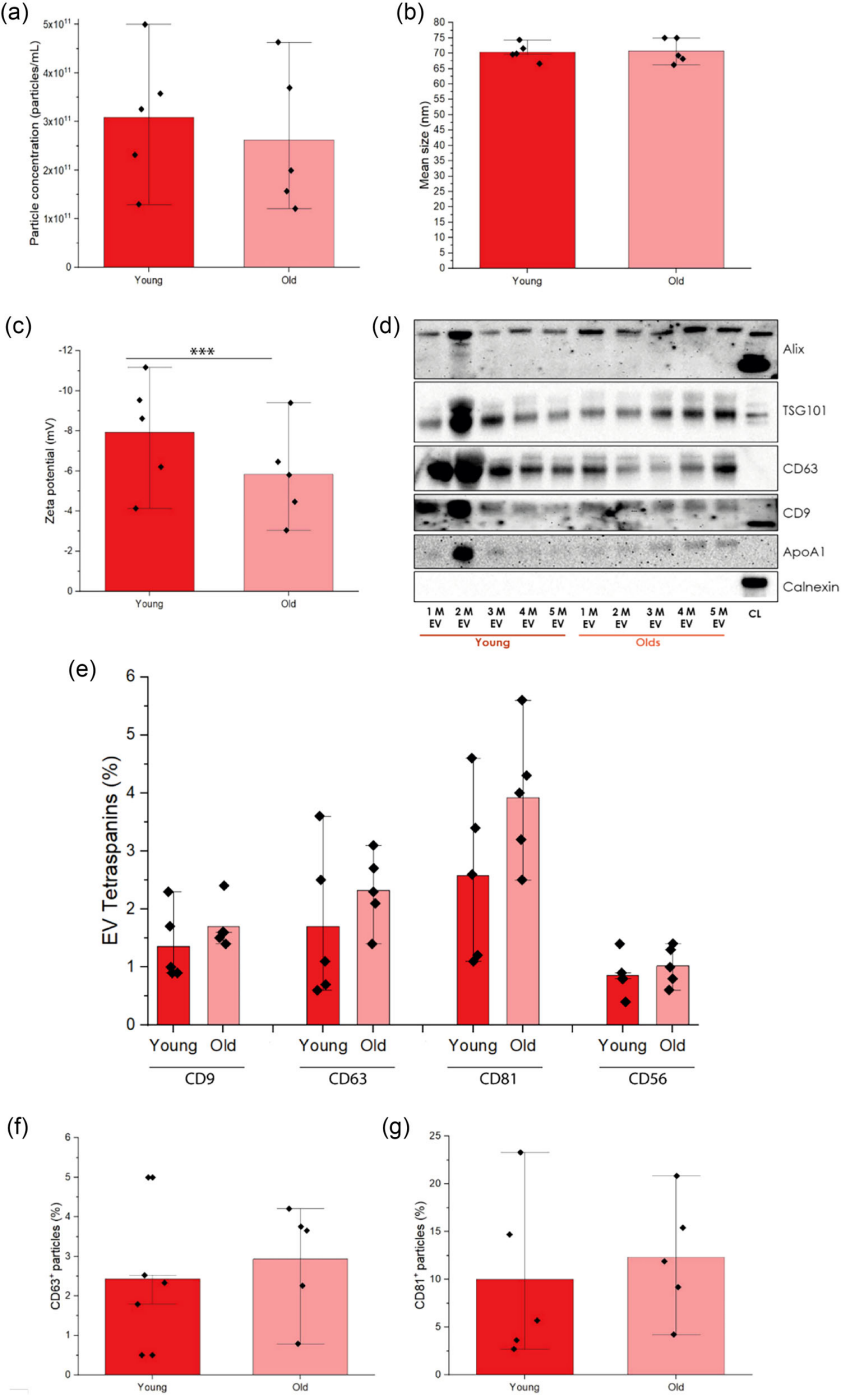
SM‐EV samples from young and old individuals’ basic characterisation, following official guidelines. (a) Particle concentration measurements for all participants included in this research. (b) Mean particle size of particles (nm) isolated per group. (c) Zeta potential measurements (mV) (***p < 0.001). (d) Positive and negative EV marker detection by Western blot (WB). Alix, TSG101, CD63 and CD9 were used to identify the presence of endosomal biogenesis and EV tetraspanins in SM‐EV samples. ApoA1 and Calnexin were used as negative markers to determine the presence of lipoproteins and ER components, respectively. (e) Nano flow cytometry (nFCM) EV tetraspanins (CD9, CD63 and CD81) and CD56 detection for young and old SM‐EVs. (f) ExoELISA results expressed as percentage populations from the total for the identification of CD63^+^ particles individually. (g) ExoELISA results expressed as percentage populations from the total for the identification of CD81^+^ particles individually average CD63^+^ composition per sample group.

To identify EV‐associated variations during SM ageing EVs were isolated using an optimised UF + SEC protocol (Fernández‐Rhodes et al., 2023) (Figure S1). Overall, no significant differences were encountered in particle concentration, size, zeta potential or EV marker profile between young and old CD56^+^ samples (Figure S2). As such, a comparison between young and old SM‐EVs in MP models was prioritised for the remainder of the paper. No significant differences in particle concentration were observed between young and old SM‐EVs (2.49 × 10^11 ^± 3.66 × 10^10^ particles/mL vs. 3.35 × 10^10 ^± 2.32 × 10^11^ particles/mL, respectively) (Figure 2a). Mean (70.44 ± 0.87 nm for young SM‐EVs vs. 70.76 ± 0.13 nm for old SM‐EVs) and mode (65.60 ± 0.59 nm for young SM‐EVs vs. 66.45 ± 0.14 nm for old SM‐EVs) (data not shown in the figure) particle sizes did not differ significantly between young and old MP samples (Figure 2b). Variations in zeta potential were evident, with SM‐EVs from older participants displaying more negative values (−8.21 ± 1.98 mV vs. −5.74 ± 0.95 mV, respectively) (*p* < 0.001) (Figure 2c).

WB was applied to detect common EV markers between SM‐EV samples (Figure 2d). Qualitative WB analysis identified the presence of described EV markers (Alix, TSG101, CD63 and CD9) in both young and older samples (Figure 2d). Minor variations could be observed in the expression of these proteins between participants but no clear qualitative variations able to be made between age groups. SM‐EVs from participant 2 displayed comparatively intense bands of all proteins analysed. ApoA1 (negative marker for high‐density lipoproteins) expression was minimal, again with the notable exception of young participant 2. Calnexin (negative marker for ER) was absent from all samples (Figure 2d). To quantify the expression of tetraspanins associated with EV biogenesis, SM‐EV samples from young and older participants were pooled and subject to nFCM analysis. CD9, CD63 and CD81 were identified in all SM‐EV samples. Total tetraspanin positivity accounted for <2% (CD9), < 3% (CD63) and <5% (CD81) of the SM‐EV population (Figure 2e). The presence of the myogenic marker CD56 was minimal for all SM‐EVs, being lower than 1.22% of the total particles (0.86 ± 0.36% for young vs. 1.2 ± 0.33% for old, *p* > 0.05) (Figure 2e). CD63 and CD81 data were further supported by ELISAs, which displayed the same trends between young and old SM‐EVs (CD63: 2.43 ± 1.45% vs. 2.64 ± 1.37%, *p* > 0.05; CD81: 10.00 ± 2.15% vs. 12.32 ± 5.58%, *p* > 0.05).

### 3.3 Biochemical profiling by Raman spectroscopy

Raman spectroscopy (RS) is a label‐free, non‐invasive method that can be applied to reveal qualitative and quantitative variations in EV biochemistry. We applied RS to profile SM‐EVs derived from SM‐EVs obtained from HPMC from young and older cohorts, measuring 500 spectra overall for each group. Again, the results displayed in the main text reflect data obtained using MP HPMC models. Similar findings were reported for CD56^+^ HPMC models (Figure S3).

**FIGURE 3:**
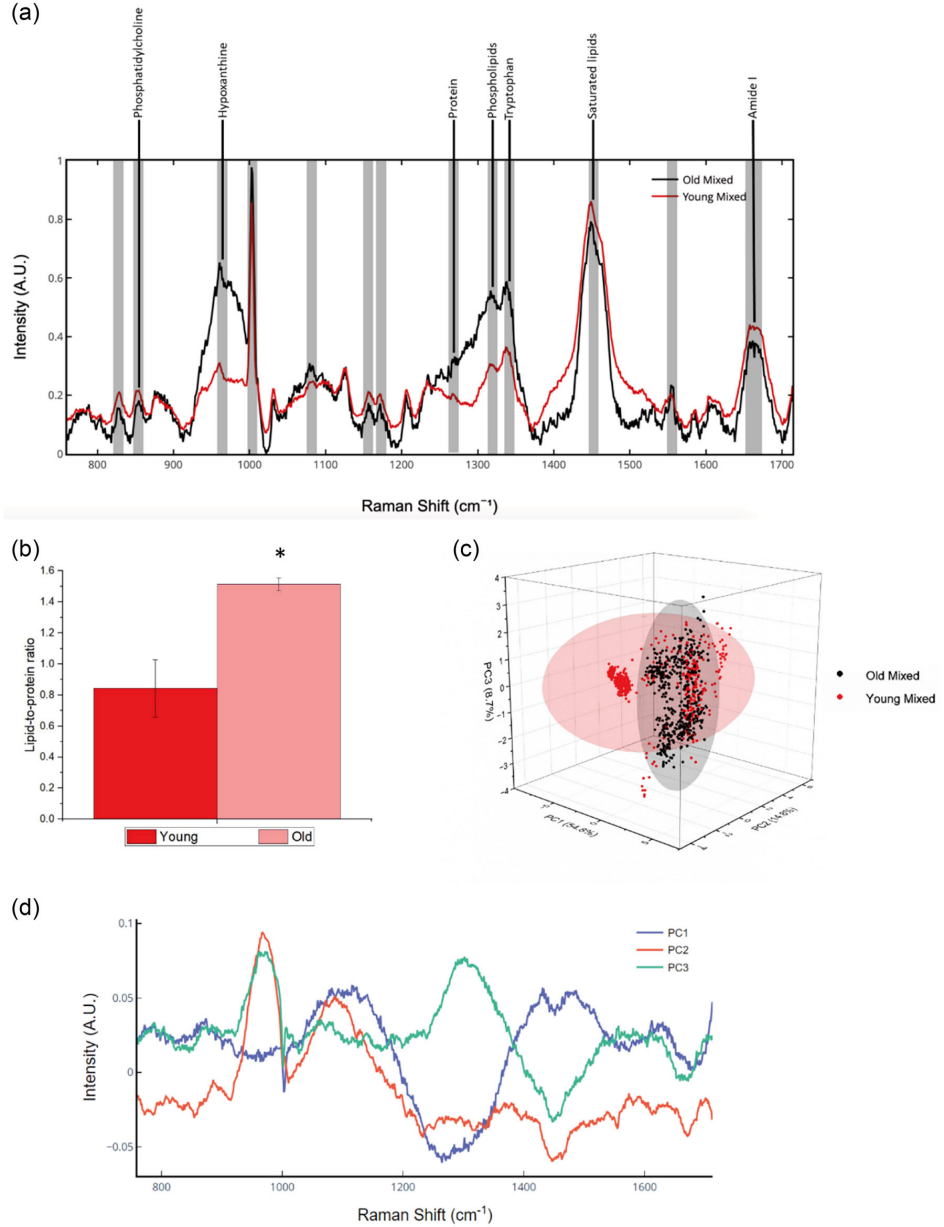
Raman spectroscopy analysis of young and old SM‐EVs. 1000 spectra overall were collected for this dataset, 500 for each group. (a) Average Raman spectra (800 to 1700 cm^−1^) representing young and old SM‐EVs with the main Raman bands highlighted with grey longitudinal lines with their corresponding assignments. (b) Lipid to protein ratios (*p < 0.05). (c) 3‐dimensional (3D) PCA analysis of young and old MP SM‐EV. (d) Representation of the loadings of the first 3 PCs. Peaks of variance at 960, 1270, 1449 and 1656 cm^−1^.

Comparisons between Raman spectra revealed that old SM‐EVs exhibited a statistically larger phosphatidylcholine (875 cm^−1^), hypoxanthine (960 cm^−1^), phospholipid (1317 cm^−1^), amide III (1250 cm^−1^) and Tryptophan signatures (*p* < 0.05), (Figure 3a). SM‐EVs isolated from older HPMCs presented an increased abundance of saturated lipids (1450 cm^−1^) compared to young SM‐EVs (*p* < 0.05) (Figure 3a). Lipid‐to‐protein ratios, based on coefficient between the peaks at 1314 cm^−1^ and 1656 cm^−1^, were significantly larger for older SM‐EV samples (0.84 ± 0.18 vs. 1.51 ± 0.04, respectively; *p* < 0.05) (Figure 3b).

PCA was used to identify major spectral changes within the dataset, presenting a PCA scores plot graph utilising 500 spectra for both young and old SM‐EVs (Figure 3c). PCA analysis revealed two differentiated clusters with overlapping interquartile ranges. The clusters of both young and old SM‐EVs revealed two distinguishable groupings with older SM‐EV sample types indicating a narrower distribution compared to young SM‐EVs. The 95% confidence ellipses of these EVs indicated a degree of sample crossover, however, based on PC1, this difference was not statistically significant. Each dataset was clustered while maximising the covariance, with the majority of the variability captured by PC1 at 54.8% and the residual captured by PC2 and PC3 at 14.8% and 8.7%, respectively. SM‐EVs from the old groups tended to narrowly cluster to the positive side of PC1, whereas the younger SM‐EVs were located on the negative side of PC1. (Figure 3c). Although there were overlaps between the components, as indicated in the PCA graph (Figure 3c), peaks displaying the most significant differences between groups were located at 960 cm^−1^ (hypoxanthine), 1317 cm^−1^ (fatty acids/tryptophan) and 1450 cm^−1^ (protein/lipid) (Figure 3d), whereas 1083 cm^−1^ (lipid/carbohydrate) and 1657 cm^−1^ (Amide I) were significant to PC2 and 1133 cm^−1^ (protein) and 1270 cm^−1^ (Amide III α‐helix) in PC3 (Figure S3). These seven peaks capture the majority of the difference between young and old SM‐EVs for sample classification.

### 3.4 Proteomic profiling by LC‐MS

**FIGURE 4:**
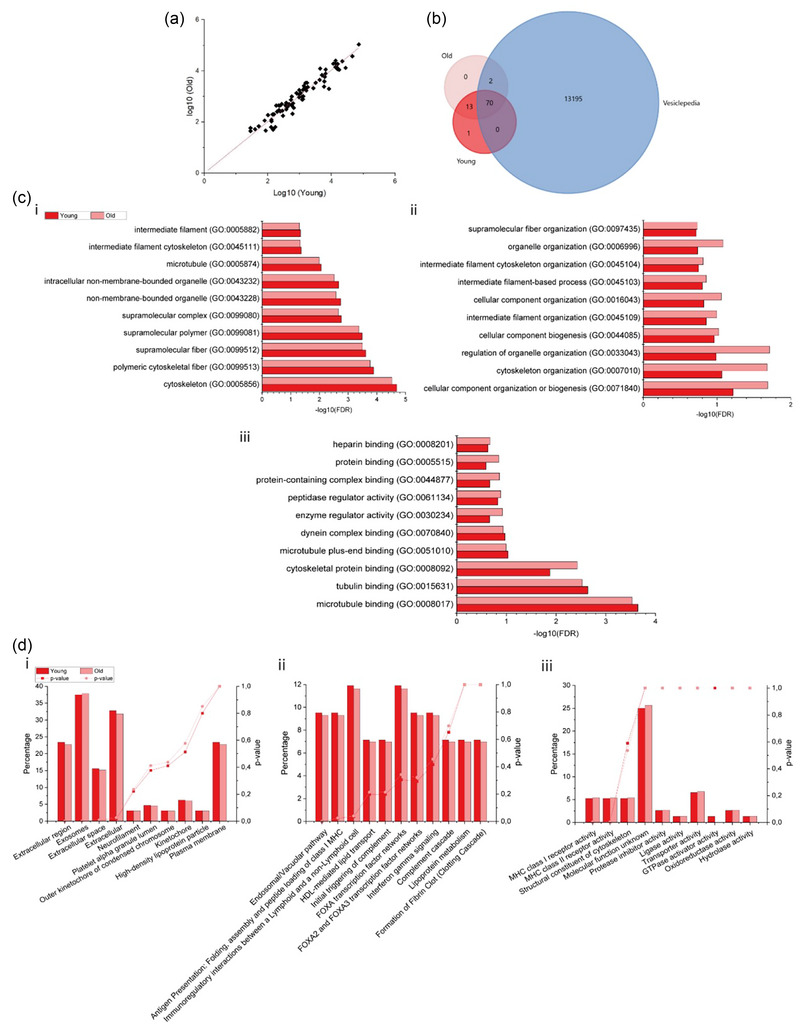
LC‐MS/MS proteome identification. (a) Linear regressions for young and old individuals derived SM‐EVs. (b) Venn diagram comparing young and old SM‐EV differences between them and the Vesiclepedia database. (c) Top10 GO‐slim analysis done using Panther for (i) ‘cellular compartment’, (ii) ‘biological process’ and (iii) ‘molecular function’. (d) FunRich analysis to display EV protein enrichment to EV literature in young and old mixes SM‐EV samples. Here we represented classification depending on (i) ‘cellular component’, (ii) ‘biological pathway’ and (iii) ‘molecular function’.

SM‐EVs were subjected to LC‐MS analysis, revealing the presence of 83 common proteins within the SM‐EV samples. Only 1 protein was unique on young MP SM‐EVs and the Vesiclepedia database, which corresponded to Cyclin‐dependent kinase‐like 3 (CDKL3). No unique proteins were identified in the old SM‐EV samples (Figure 4b). Linear regression revealed a correlation of over 99% (*R* ^2 ^= 0.99442) in protein abundance (Figure 4a). Comparing both proteomes against Vesiclepedia, 70 common proteins were identified as EV associated, accounting for 84% of those identified. These included HLA complexes, Serpins and membrane proteins. The full list of common EV proteins can be found in Vesiclepedia, Study ID: 3591 (Chitti et al., 2024) None of these proteins were absent in the young SM‐EVs. However, 2 were uniquely related to SM‐EVs in the old samples but not registered in the Vesiclepedia database. These proteins were complement C4‐A (C4), and Ras GTPase activating like protein IQGAP2 (IQGAP2). PANTHER GO protein classification organised the shared proteins in three different GO categorizations, ‘cellular compartment’, ‘biological processes’ and ‘molecular functions’ (Figure 4c). Cellular compartment categorisation revealed proteins associated with cytoskeletal structures (GO:0005856, GO:0099513, GO:0045111), intermediate filament (GO:0005882) or non‐membranal organelles (GO:0043228, GO:0043232) (Figure 4 c(i)). Biological processes corresponded with organelle organisation (GO:0071840, GO:0033043, GO:0016043) cellular component biogenesis (GO:0044085, GO:0006996) and cytoskeleton and related structures organisation (GO:0007010, GO:0045109, GO:0045103, GO:0045104, GO:0097435) (Figure 4c(ii)). Molecular function identified proteins related to microtubule and tubulin binding (GO:0051010, GO:0070840, GO:0008017, GO:0015631), enzymatic regulation (GO:0061134, GO:0030234) and protein binding (GO:0044877, GO:0008201, GO:0005515) (Figure 4c(iii)).

Finally, SM‐EV proteomes were analysed using Functional Enrichment Analysis (FunRich) (Figure 4d). For both young and older SM‐EV samples, cellular component classification revealed that significantly larger protein percentages were related to ‘extracellular region’, ‘exosomes’, ‘extracellular space’ and ‘extracellular’ (Figure 4d(i)). Significantly upregulated biological pathways were linked to endosomal pathways and immunogenicity. Non‐significant but larger associations were also found in lipid and lipoprotein and glucose metabolism (Figure 4d(ii)). Finally, molecular function classification revealed significantly upregulated MHC receptor activities and structural constituent of the cytoskeleton. (Figure 4d(iii)).

**FIGURE 5:**
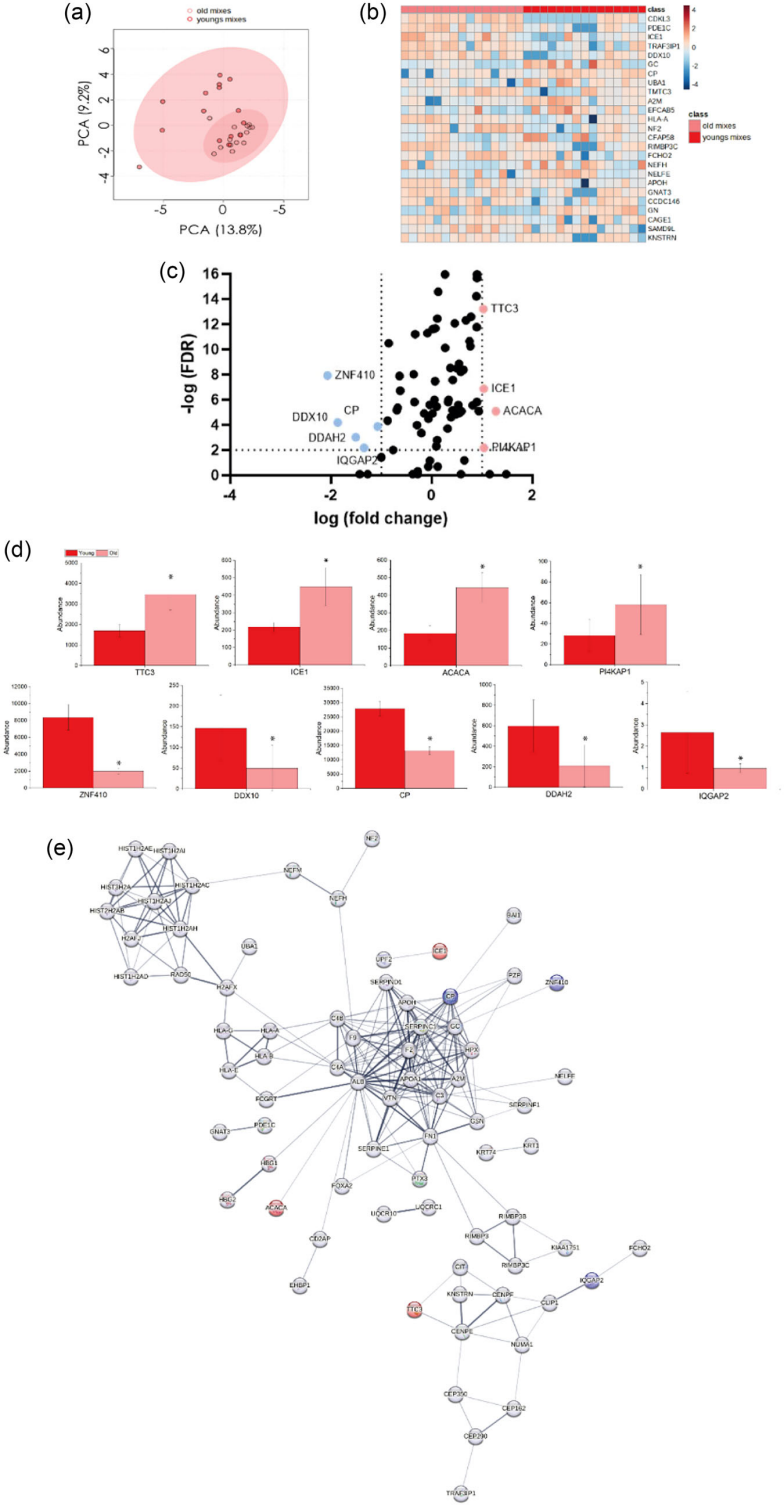
Comparative analysis of proteins from young and old SM‐EVs. (a) PCA component analysis for young and old SM‐EV groups. (b) Heatmap representing top 25 proteins. (c) Volcano plots displaying Log_2_ values for protein fold‐change against Log_10_ false discovery rate (FDR). Only proteins with a Log_2_ fold change of >1 and a *p* < 0.05 were considered to be statistically significant. Upregulated proteins were indicated in red and downregulated in blue. (d) Bar graphs indicating differences in abundance for significantly up and downregulated proteins. *p < 0.05. (e) STRING analysis. Whole proteome was represented. Moreover, upregulated proteins were indicated in red and downregulated in blue.

Protein abundance data for both young and old HPMC‐derived SM‐EVs was submitted to Metaboanalyst to identify patterns in their differential expression (Figure 5). PCA plots showed cluster or group separation—although overlap was observed. Clustering within the old SM‐EV sample population was visually more homogenous (Figure 5a). PC1 (13.8%) and PC2 (9.2%) reflected only low variation in differential protein expression between groups (Figure 5a). Heatmap representation of the top 25 proteins clustered visualising patterns in sample groups and individual participants revealed the most variable protein is CDKL3, followed by factors such as ICE1 and DDX10 (Figure 5b). Volcano plots identified significant (Log_2_ FC≥1, *p* < 0.05) upregulation in 4 proteins within the older participant group: E3 ubiquitin‐protein ligase TTC3 (TTC3), Little elongation complex subunit 1 (ICE1), Acetyl‐CoA carboxylase 1 (ACACA) and Putative inactive phosphatidylinositol 4‐kinase alpha‐like protein P1 (PI4K4P1). Older SM‐EVs also contained 5 significantly downregulated proteins: Zinc finger protein 410 (ZNF410), ATP‐dependent RNA helicase DDX10 (DDX10), N(G),N(G)‐dimethylarginine dimethylaminohydrolase 2 (DDAH2), Ras GTPase activating like protein IQGAP2 (IQGAP2) and ceruloplasmin (CP) (Figure 5c,d). STRING analysis (Figure 5e) supported results obtained via PANTHER and FunRich analysis. Significantly upregulated and downregulated proteins were indicated in the networks using the same colour coding applied in the volcano plots (young SM‐EV in blue and older SM‐EV in red). Represented proteins related to three main clusters: cellular maintenance, energy expenditure—including glucose metabolism, Ca^2+^ (CP)—and fatty acid regulation (ACACA) and proteasome and transcriptome homeostasis (TTC3, IQGAP1 and ICE1) (Figure 5e).

## 4 DISCUSSION

Despite a growing understanding of the importance of EVs in myogenesis, SM‐EV changes associated with ageing remain largely undefined in human models. The present study applied an optimised SM‐EV isolation protocol offering enhanced levels of purity (Fernández‐Rhodes et al., 2023), to deliver the first comparative profile of EV variations occurring during SM ageing in an established primary human myogenic model (Fleming et al., 2020; Rimington et al., 2021). Our data indicated a newly described role for SM‐EVs in providing an alternative homeostasis and detoxification pathway during myoblast ageing. Outcomes provide a novel insight into the function of EVs in SM biology that is distinct from their previously acknowledged roles in autocrine and paracrine communication.

In line with previous observations, our microscopic data identified a trend towards reduced myotube fusion and differentiation in HPMCs isolated from older donors (Figure 1, Figure S1), with SM ageing known to induce variations in MyHC expression and the spatial distribution of fibres (Cristea et al., 2010; Larsson et al., 2019). Similar results have been presented in engineered murine SM models generated using cultures that had undergone multiple population‐doublings (Sharples et al., 2012). These trends appear to be consistent irrespective of the SM model applied. However, current understanding of possible variations in SM‐EV profiles associated with SM ageing have largely been confined to murine models, with only limited research emerging from human systems. Observations from murine models have highlighted variations in the EV proteome and transcriptome during SM ageing, with EVs isolated from aged mice outnumbering those from young (Sahu et al., 2021). Interestingly, EVs from older mice also contained significantly less Klotho transcripts, myomiRNAs (miR‐1, mR‐133a, miR‐133b and miR‐206) and miRNAs (miR‐34a and miR‐690), which collectively represent a mixture of postulated and well‐defined regulators of SM metabolism and ageing (Fulzele et al., 2019; Mytidou et al., 2021; Sahu et al., 2021; Shao et al., 2022). In the single human study currently published on SM ageing, prematurely aged HPMC models induced by hydrogen peroxide (H_2_O_2_) exposure produced significantly higher concentrations of SM‐EVs, including a 5‐fold increase of EVs in the exosome size range, when isolated using a commercial precipitation kit. The EV containing fractions recovered were principally associated with inflammatory factors such as TGF‐β and MMP2, with potential implications for the dysregulation of the myoblast cell cycle, inflammation and paracrine senescence events associated with endothelial cells (Hettinger et al., 2021). However, while these findings are of interest, there are several limitations to this study. These limitations relate to the ageing model applied and the application of a commercial precipitation reagent for EV isolation. The application of H_2_O_2_ is limited in its ability to accurately model ageing. Rather, H_2_O_2_ induces a rapid increase of reactive oxygen species (ROS), inflammation and the induction of apoptosis in C2C12 (Kinoshita et al., 2019) and human satellite cells and myoblasts (Fei et al., 2015; Sies & Jones, 2020). Research presented in our study applied HPMCs from aged individuals to provide an improved approach to study SM ageing in vitro. EV isolation methods applied in previous human SM studies, not exclusively focused on SM ageing, have principally utilised commercial isolation kits or differential ultracentrifugation (dUC). These methods will provide EV enriched fractions, as reflected by the presence of EV specific markers such as Alix and tetraspanins (Choi et al., 2016; Fry et al., 2017; Hettinger et al., 2021; Huang et al., 2023; Le Bihan et al., 2012). However, they also result in the co‐isolation of many non‐EV contaminants such as protein/lipid aggregates and lipoproteins with overlapping densities (high density lipoproteins: 1.06 – 1.21 g/mL) and diameters (low density lipoproteins: 20 – 200 nm) (Taylor & Shah, 2015; Yuana et al., 2014). As such, the application of such non‐specific protocols can reduce sample purity and introduce non‐EV contaminants that can confound downstream data analysis and potential misinterpretation of contribution of EVs to myogenic processes. For example, it is well described that lipoproteins can function as mRNA carriers (Karvinen et al., 2023; Vickers et al., 2011) and vastly outnumber EVs in biofluids such as plasma (Guescini et al., 2015). Consequently, the recovery of lipoproteins in SM‐EV samples may confound the interpretation of the contribution of EVs in myogenesis and SM ageing. SM‐EV preparations recovered in the present study displayed low lipoprotein contamination (Figure 2, Figure S2) due to the specificity of the optimised isolation protocol applied (Fernández‐Rhodes et al., 2023). In line with previous publications, ApoA was the selected marker to investigate the presence of high‐density lipoproteins in our preparations. In our previous work, we demonstrated that ApoB^+^ particles were typically absent in SM‐EV isolations (Fernández‐Rhodes et al., 2023). However, the presence of lipoprotein recovery in in some of our preparations (e.g., young participant 2) may indeed impact the interpretation of some factors, such as zeta potential (ZP) (Belle et al., 1997; Sparks et al., 2008). While ZP values for SM‐EVs have not previously been reported in the literature. Values recorded in the present study were typically less negative compared to those previously reported for EVs from other sources (Midekessa et al., 2020; Varshosaz et al., 2012; Wilson & Green, 2017). It should also be noted that ZP measurements are also influenced by storage times and temperatures (Gelibter et al., 2022; Maroto et al., 2017) and conditions such as pH or salts concentration (Midekessa et al., 2020), which are often not consistent between publications. Furthermore, we were able to validate the expression of tetraspanin surface proteins (CD9, CD63 and CD81) associated with EV biogenesis for the first time in SM‐EV preparations at the single EV level using high resolution nFCM (Figure 2). The abundance of EV tetraspanins and other EV markers in SM‐EVs has previously only been reported using semi‐quantitative techniques, such as WB (Alibhai et al., 2020; Xhuti et al., 2023), or by mass spectrometry (Forterre et al., 2014; Le Bihan et al., 2012). The latter technique, while quantitative, cannot distinguish between EVs and co‐isolated proteins. While we could detect the presence of tetraspanins among all samples tested, they were not abundant. Similar observations have been noted in EV preparations from other cell sources, with heterogeneity in their expression reported across different cell sources and outcomes dependent on the sensitivity of the method of analysis applied (Bortot et al., 2022; Céspedes et al., 2022; Kowal et al., 2016–Mizenko et al., 2021). For example, MSCs derived from bone marrow (BM‐MSCs), adipose (ADSCs) and umbilical cord MSCs (UC‐MSCs) presented notable variations in CD63 and CD9 expression (Gualerzi et al., 2017; Skovronova et al., 2021). Tetraspanin profiles have also been found to vary based in parameters such as EV biogenesis and size, with smaller exosomes (≤ 150 nm) typically having an increased expression (Lischnig et al., 2022). As such, further investigation may be required to evaluate the relative distribution of tetraspanins on SM‐EVs. Lastly, future studies must take into consideration growing evidence on the potentially negative impact of sample storage on surface protein expression before any clear conclusions can be made (Görgens et al., 2022; Yuan et al., 2021). Based on these recent observations, we acknowledged this as a limitation in the present article, which would require a deeper observation of tetraspanin expression differences overtime, and the broader SM‐EV field.

Due to an absence of notable variations in standard EV characteristics, we applied RS as sensitive, label‐free method to comprehensively profile qualitative and quantitative variations in the biochemical signatures of SM‐EVs (Figures 2 and 3). RS has previously been applied in EV research to distinguish the presence of lipoprotein signatures common to EV preparations (Enciso‐Martinez et al., 2020; Koster et al., 2021; Zini et al., 2022), to profile tissue‐specific differences in MSC‐EV composition (Gualerzi et al., 2017) and to observe temporal changes in EV biochemistry during myogenesis (Davies et al., 2021). A previous study found that serum EVs from aged individuals displayed a decrease in nucleic acid composition and an increase in lipid signatures when compared with young individuals (Sahu et al., 2021). Similar findings were recorded in the present study for SM‐EV samples obtained from older donors with higher intensity peaks observed for phospholipids (Figure 3). Parlatan and colleagues previously showed that a Raman shift between 970 and 1100 cm^−1^ represented the primary EV signature, identifying nucleic acids and phospholipids common to these preparations (Parlatan et al., 2023). In our study, spectra obtained from young and old SM‐EV samples displayed clear biochemical variations within this window, with variations in phosphatidylcholine (PC) (875 cm^−1^) and hypoxanthine (960 cm^−1^) identified (Figure 3). Variations in EV phospholipid profiles have previously been associated with pathophysiological conditions including neurodegenerative diseases and cancer (Chang et al., 2022). For example, EVs secreted from drug‐resistant lung cancer cells presented an increase in PC (Jung et al., 2015). Within SM studies, negative associations have been reported between certain phospholipids (PC, PE and PG) and muscle volume (Uchitomi et al., 2019). Some have reported changes in phospholipid composition can be related to EV size, with exosome‐like vesicles typically containing increased quantities of PC (Zhang et al., 2018). However, we saw no such relationship in our study. This information could be complemented in the future by developing lipidomics or metabolomis studies of these samples, as it has been reported to describe EVs in neurodegeneration or cancer (Liu et al., 2023; Shaba et al., 2022; Su et al., 2024). In addition to observed variations in phospholipid content, RS also highlighted an increased presence of hypoxanthine within SM‐EV samples from older participants. Hypoxanthine is a purine derivative generated during adenine deamination that can lead to DNA mutagenesis (DeVito et al., 2017). An increased association between SM‐EVs from older HMPCs and hypoxanthine is an intriguing finding as it suggests that EVs could be utilised in transcriptomic regulation to reduce the likelihood of DNA mutagenesis in ageing myoblasts—a process that is associated with senescence and apoptosis (Budke & Kuzminov, 2006; Davies et al., 2012). Furthermore, this finding also harkens back to an initial proposed role for EVs as so‐called ‘garbage bags’ of the cell that principally function in the removal of waste and deleterious materials (Vidal, 2019). The presence of intermediate metabolites of purine/pyrimidine metabolism has previously been reported for multiple cancer‐derived EVs, which were able to exhibit an immunosuppressive effect that may facilitate immune escape and cancer metastasis (Ludwig et al., 2020; Tadokoro et al., 2020). Within SM, an increased presence of hypoxanthine could also signify a possible role for SM‐EVs in detoxification during myoblast ageing, with the metabolism of hypoxanthine during SM contraction known to lead to the generation of ROS (Bouviere et al., 2021). Interestingly, Brault et al. (2001) indicated that hypoxanthine may escape the myocyte through the plasma membrane (Brault & Terjung, 2001). However, to the best of our knowledge, the present study is the first to associate the release of hypoxanthine from HPMCs with EVs.

RS outputs reflected variations in protein content between SM‐EVs from young and older participants (protein: 1270, amide I: 1656 cm^−1^) and proposed a possible role for SM‐EVs in HPMC waste removal and detoxification during ageing. Therefore, we next sought to compare age‐related variations in the SM‐EV proteome using LC‐MS. The majority of proteins (98.8%, *R* ^2^ value of 0.99442) were common between young and old SM‐EVs, with only a small number of unique proteins detected. Unique proteins identified in old SM‐EVs included C4—an element of the classical complement pathway (Legoedec et al., 1997)—and Ras GTPase activating like protein IQGAP2 (IQGAP2)—a regulator in glycogen metabolism (Sen et al., 2023). However, multiple proteins associated with proteomic/transcriptomic regulation and oxidative stress were found to be differentially expressed (Figures 4 and 5). These outcomes complement our RS data in describing a possible novel role for SM‐EVs in cellular detoxification during ageing, with oxidative stress being one of the hallmarks of this process (Jackson et al., 2022; Lian et al., 2022; McArdle & Jackson, 2019). Within SM‐EV isolates from older HPMCs, several proteins crucial for SM signalling and metabolic regulation were significantly downregulated (Figure 5). This included IQGAP1, which is involved in glycogen metabolism, insulin regulation and cell signalling (Sen et al., 2023; Smith et al., 2015). We also identified an upregulation of the E3 ubiquitin ligase TTC3 in SM‐EVs obtained from aged HPMCs. Although limited studies have focused on the roles of E3 ubiquitin ligases in this process, researchers have observed that some E3 ligases (e.g., CRL family members) are transcriptionally more active in instances of SM wasting (Bodine et al., 2001; Lee et al., 2016). Acetyl‐CoA Carboxylase Alpha (ACACA) was also increased in older SM‐EVs. This enzyme catalyses a rate‐limiting step in fatty acid synthesis and has been broadly linked to the presence of oxidative stress (Davies et al., 2021; Ichimura et al., 2015; Muratore et al., 2012–Takagi et al., 2021). Together with RS data (Figure 3), an increased presence of ACACA could reflect previously reported age‐related shifts in phospholipid content in ageing SM (Lee et al., 2020). Lastly, ATP‐dependent RNA helicase DDX10 was downregulated in SM‐EV samples from older individuals. The involvement of DDX10 in ribosome biogenesis (Sergeeva & Zatsepin, 2021), similarly to other helicases from the same family such as DDX27, could indicate a modulation of Pax7 in myogenesis and oxidative equilibrium (Bennett et al., 2018). Lack of balance of these helicases may cause RNA mutagenesis and toxicity.

A possible limitation of the present study is a lack of miRNA profiling. MyomiRNAs, miR‐1, mR‐133a, miR‐133b and miR‐206, have been associated with exercise, disease and SM ageing (De Gasperi et al., 2017; Estébanez et al., 2021; Mytidou et al., 2021; Matsuzaka et al., 2016; Castaño et al., 2020). In fact, Mytidou and colleagues showed that miR‐1, miR‐133a and miR‐133b levels were increased during SM ageing, suggesting a role in SM homeostasis (Mytidou et al., 2021). However, some controversy concerning the real importance and effects of miRNA associated with EVs have recently started to arise, owing to their potentially low copy numbers (around 1 miRNA molecule per 100 EVs) and limited cellular delivery (Albanese et al., 2021). A limited amount of evidence exists to support these observations in SM. This is perhaps best evidenced by the fact that myomiRNAs miR‐1, miR‐133a and miR‐206 were shown to be present at less than one copy per 100 EVs (Hanson et al., 2023). Furthermore, a life‐long reduction of myomiRNA expression was found not to affect SM morphology in murine models (Vechetti et al., 2019), questioning the criticality of their contributions to myogenesis. Nonetheless, further research is required to fully evaluate the prevalence and functional contribution of EV‐associated miRNAs in SM physiology and ageing. In addition to these studies, the SM‐EV isolation protocols developed in this manuscript lay the groundwork for studying SM‐EVs in the contexts of exercise and ageing using increasingly complex models. Thus, validating these models is essential if future research on 3D SM models is to incorporate HPMCs. These bioengineered tissues demonstrated the ability to undergo muscle regeneration, replicating key processes of native SM. Furthermore, combining CD56^+^ and CD56^−^ populations has been shown to create a robust bioengineered 3D SM model with differentiated myotubes and regenerative capabilities (Fleming et al., 2020). This bioengineered system has also been co‐cultured with motor neuron progenitors to develop an NMJ model (Rimington et al., 2021), showcasing its versatility and potential for application in SM‐EV research in ageing and disease.

## 5 CONCLUSION

**FIGURE 6:**
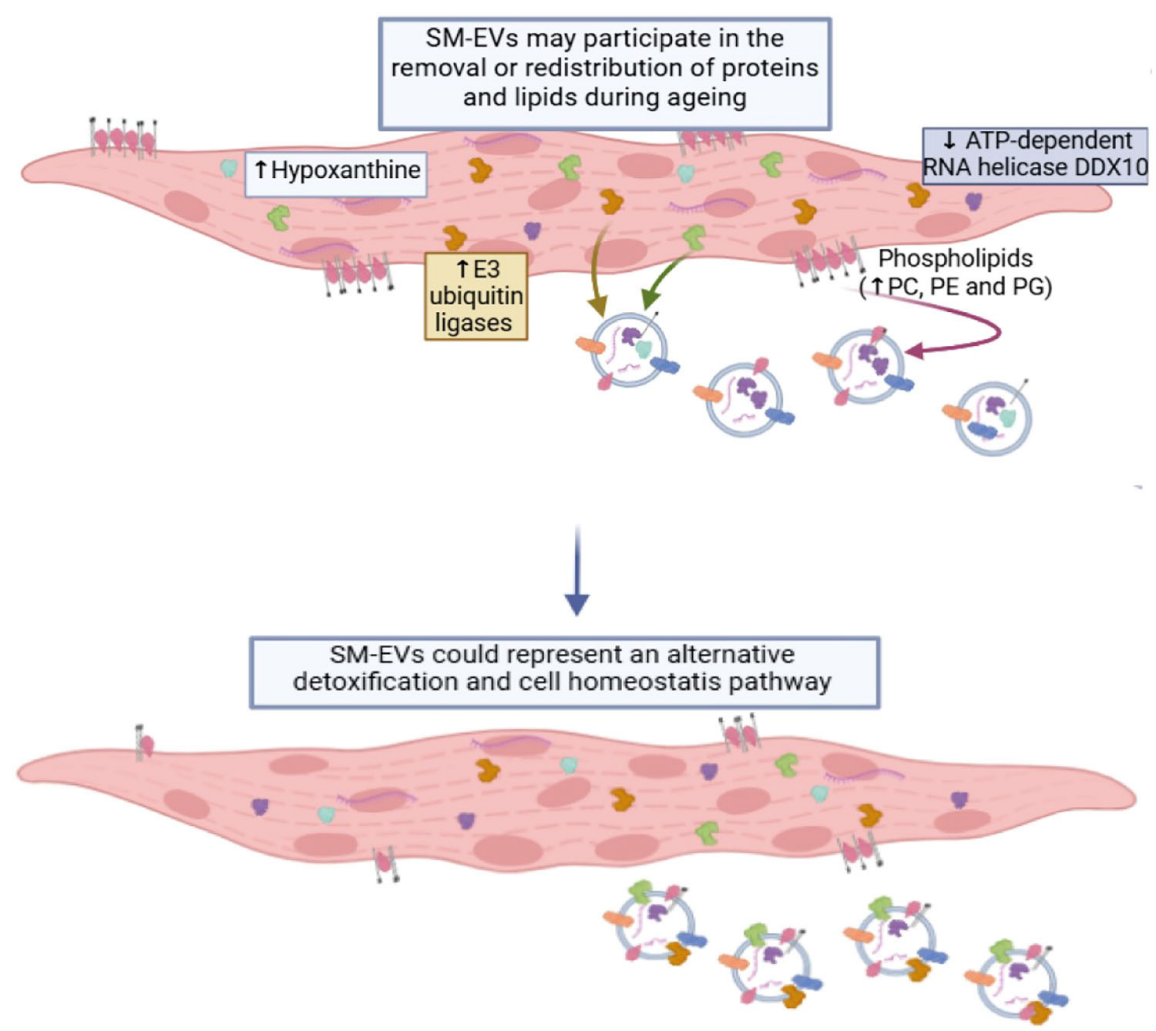
Overview figure. SM cells experience during ageing the accumulation or unbalanced removal of factors, which are related to muscle functioning, oxidative stress, DNA mutagenesis or cellular maintenance. This dysregulation could lead to a fastest ageing process. We suggested that SM‐EVs might be alleviating those effects by acting as alternative detoxification or homeostasis tools, controlling the presence of those dysbalanced factors by facilitating their secretion or regulating their intracellular presence.

Through the application of an optimised SM‐EV isolation protocol, our study revealed minimal variation in basic EV parameters, such as concentration and EV tetraspanin expression between young and old SM‐EVs samples. However, detailed biochemical and proteomic comparison of the SM‐EV content revealed a differential expression of phospholipids, hypoxanthine and proteins broadly implicated in maintaining proteome homeostasis, SM maintenance and detoxification. In conclusion, SM‐EV composition could suggest a window into age‐related changes in SM. SM‐EV role in ageing SM might be linked to the removal or redistribution of dysfunctional factors as an alternative detoxification and homeostasis pathway (Figure 6).

## AUTHOR CONTRIBUTIONS

**María Fernández‐Rhodes**: Data curation (lead); formal analysis (lead); investigation (equal); methodology (equal); writing—original draft (lead). **Emma Buchan**: Formal analysis (supporting); resources (supporting). **Stephanie D. Gagnon**: Methodology (supporting); resources (supporting). **Jiani Qian**: Methodology (supporting); resources (supporting). **Lee Gethings**: Formal analysis (supporting); resources (supporting); software (supporting). **Rebecca Lees**: Formal analysis (supporting); methodology (supporting); resources (supporting). **Ben Peacock**: Formal analysis (supporting); methodology (supporting); resources (supporting). **Andrew J. Capel**: Investigation (supporting); project administration (supporting); supervision (supporting); writing—review and editing (supporting). **Neil R. W. Martin**: Methodology (supporting); resources (supporting). **Pola Goldberg Oppenheimer**: Data curation (supporting); resources (supporting). **Mark P. Lewis**: Conceptualization (supporting); writing—review and editing (supporting). **Owen G. Davies**: Conceptualization (lead); funding acquisition (lead); investigation (equal); project administration (lead); supervision (lead); writing—review and editing (lead).

## CONFLICT OF INTEREST STATEMENT

The authors declare no conflicts of interest.

## Supporting information

Supporting Information

## Data Availability

The data that support the findings of this study are openly available in Vesiclepedia at http://microvesicles.org/index.html, reference number 3591.

## References

[jex2171-bib-0001] Albanese, M. , Chen, Y. A. , Hüls, C. , Gärtner, K. , Tagawa, T. , Mejias‐Perez, E. , Keppler, O. T. , Göbel, C. , Zeidler, R. , Shein, M. , Schütz, A. K. , & Hammerschmidt, W. (2021). MicroRNAs are minor constituents of extracellular vesicles that are rarely delivered to target cells. PLoS Genetics, 17(12), e1009951. 10.1371/journal.pgen.1009951 34871319 PMC8675925

[jex2171-bib-0002] Alfonzo, M. C. , Al Saedi, A. , Fulzele, S. , & Hamrick, M. W. (2022). Extracellular vesicles as communicators of senescence in musculoskeletal aging. JBMR Plus, 6(11), e10686. 10.1002/jbm4.10686 36398109 PMC9664547

[jex2171-bib-0003] Alibhai, F. J. , Lim, F. , Yeganeh, A. , DiStefano, P. V. , Binesh‐Marvasti, T. , Belfiore, A. , Wlodarek, L. , Gustafson, D. , Millar, S. , Li, S. H. , Weisel, R. D. , Fish, J. E. , & Li, R. K. (2020). Cellular senescence contributes to age‐dependent changes in circulating extracellular vesicle cargo and function. Aging Cell, 19(3), 1–14. 10.1111/acel.13103 PMC7059145 31960578

[jex2171-bib-0004] Aswad, H. , Jalabert, A. , & Rome, S. (2016). Depleting extracellular vesicles from fetal bovine serum alters proliferation and differentiation of skeletal muscle cells in vitro. BMC Biotechnology, 16, 32. 10.1186/s12896-016-0262-0 27038912 PMC4818850

[jex2171-bib-0005] Baci, D. , Chirivì, M. , Pace, V. , Maiullari, F. , Milan, M. , Rampin, A. , Somma, P. , Presutti, D. , Garavelli, S. , Bruno, A. , Cannata, S. , Lanzuolo, C. , Gargioli, C. , Rizzi, R. , & Bearzi, C. (2020). Extracellular vesicles from skeletal muscle cells efficiently promote myogenesis in induced pluripotent stem cells. Cells, 9(6), 1527. 10.3390/cells9061527 32585911 PMC7349204

[jex2171-bib-0006] La Belle, M. , Blanche, P. J. , & Krauss, R. M. (1997). Charge properties of low density lipoprotein subclasses. Journal of Lipid Research, 38(4), 690–700. 9144084

[jex2171-bib-0007] Bennett, A. H. , O'Donohue, M. F. , Gundry, S. R. , Chan, A. T. , Widrick, J. , Draper, I. , Chakraborty, A. , Zhou, Y. , Zon, L. I. , Gleizes, P. E. , Beggs, A. H. , & Gupta, V. A. (2018). RNA helicase, DDX27 regulates skeletal muscle growth and regeneration by modulation of translational processes. PLoS Genetics, 14(3), 1–25. 10.1371/journal.pgen.1007226 PMC5843160 29518074

[jex2171-bib-0008] Bodine, S. C. , Stitt, T. N. , Gonzalez, M. , Kline, W. O. , Stover, G. L. , Bauerlein, R. , Zlotchenko, E. , Scrimgeour, A. , Lawrence, J. C. , Glass, D. J. , & Yancopoulos, G. D. (2001). Akt/mTOR pathway is a crucial regulator of skeletal muscle hypertrophy and can prevent muscle atrophy in vivo. Nature Cell Biology, 3(11), 1014–1019. 10.1038/ncb1101-1014 11715023

[jex2171-bib-0009] Bortot, B. , Mangogna, A. , Peacock, B. , Lees, R. , Valle, F. , Brucale, M. , Tassinari, S. , Romano, F. , Ricci, G. , & Biffi, S. (2022). Platelet activation in ovarian cancer ascites: Assessment of GPIIb/IIIa and PF4 in small extracellular vesicles by nano‐flow cytometry analysis. Cancers, 14(17), 4100. 10.3390/cancers14174100 36077635 PMC9454670

[jex2171-bib-0010] Bouviere, J. , Fortunato, R. S. , Dupuy, C. , Werneck‐de‐Castro, J. P. , Carvalho, D. P. , & Louzada, R. A. (2021). Exercise‐stimulated ROS sensitive signaling pathways in skeletal muscle. Antioxidants (Basel, Switzerland), 10(4), 1–21. 10.3390/antiox10040537 PMC8066165 33808211

[jex2171-bib-0011] Brault, J. J. , & Terjung, R. L. (2001). Purine salvage to adenine nucleotides in different skeletal muscle fiber types. Journal of Applied Physiology (Bethesda, Md.: 1985), 91(1), 231–238. 10.1152/jappl.2001.91.1.231 11408435

[jex2171-bib-0012] Budke, B. , & Kuzminov, A. (2006). Hypoxanthine incorporation is nonmutagenic in Escherichia coli. Journal of Bacteriology, 188(18), 6553–6560. 10.1128/JB.00447-06 16952947 PMC1595496

[jex2171-bib-0013] Castaño, C. , Mirasierra, M. , Vallejo, M. , Novials, A. , & Párrizas, M. (2020). Delivery of muscle‐derived exosomal miRNAs induced by HIIT improves insulin sensitivity through down‐regulation of hepatic FoxO1 in mice. PNAS, 117, 30335–30343. 33199621 10.1073/pnas.2016112117 PMC7720135

[jex2171-bib-0014] Céspedes, P. F. , Jainarayanan, A. , Fernández‐Messina, L. , Valvo, S. , Saliba, D. G. , Kurz, E. , Kvalvaag, A. , Chen, L. , Ganskow, C. , Colin‐York, H. , Fritzsche, M. , Peng, Y. , Dong, T. , Johnson, E. , Siller‐Farfán, J. A. , Dushek, O. , Sezgin, E. , Peacock, B. , Law, A. , … Dustin, M. L. (2022). T‐cell trans‐synaptic vesicles are distinct and carry greater effector content than constitutive extracellular vesicles. Nature Communications, 13(1), 3460. 10.1038/s41467-022-31160-3 PMC9203538 35710644

[jex2171-bib-0015] Chang, W. , Xiao, D. , Fang, X. , & Wang, J. (2022). Phospholipids in small extracellular vesicles: Emerging regulators of neurodegenerative diseases and cancer. Cytotherapy, 24(2), 93–100. 10.1016/j.jcyt.2021.09.013 34742629

[jex2171-bib-0016] Chitti, S. V. , Gummadi, S. , Kang, T. , Shahi, S. , Marzan, A. L. , Nedeva, C. , Sanwlani, R. , Bramich, K. , Stewart, S. , Petrovska, M. , Sen, B. , Ozkan, A. , Akinfenwa, M. , Fonseka, P. , & Mathivanan, S. (2024). Vesiclepedia 2024: An extracellular vesicles and extracellular particles repository. Nucleic Acids Research, 52(D1), D1694–D1698. 10.1093/nar/gkad1007 37953359 PMC10767981

[jex2171-bib-0017] Choi, J. S. , Yoon, H. I. , Lee, K. S. , Choi, Y. C. , Yang, S. H. , Kim, I. S. , & Cho, Y. W. (2016). Exosomes from differentiating human skeletal muscle cells trigger myogenesis of stem cells and provide biochemical cues for skeletal muscle regeneration. Journal of Controlled Release: Official Journal of the Controlled Release Society, 222, 107–115. 10.1016/j.jconrel.2015.12.018 26699421

[jex2171-bib-0018] Conkright, W. R. , Beckner, M. E. , Sterczala, A. J. , Mi, Q. , Lovalekar, M. , Sahu, A. , Krajewski, K. T. , Martin, B. J. , Flanagan, S. D. , Greeves, J. P. , O'Leary, T. J. , Wardle, S. L. , Ambrosio, F. , & Nindl, B. C. (2022). Resistance exercise differentially alters extracellular vesicle size and subpopulation characteristics in healthy men and women: An observational cohort study. Physiological Genomics, 54(9), 350–359. 10.1152/physiolgenomics.00171.2021 35816651

[jex2171-bib-0019] Cristea, A. , Qaisar, R. , Edlund, P. K. , Lindblad, J. , Bengtsson, E. , & Larsson, L. (2010). Effects of aging and gender on the spatial organization of nuclei in single human skeletal muscle cells. Aging Cell, 9(5), 685–697. 10.1111/j.1474-9726.2010.00594.x 20633000

[jex2171-bib-0020] Davies, O. , Mendes, P. , Smallbone, K. , & Malys, N. (2012). Characterisation of multiple substrate‐specific (d)ITP/(d)XTPase and modelling of deaminated purine nucleotide metabolism. BMB Reports, 45(4), 259–264. 10.5483/bmbrep.2012.45.4.259 22531138

[jex2171-bib-0021] Davies, O. G. , Powell, S. , Rickard, J. J. , Clancy, M. , & Goldberg Oppenheimer, P. (2021). Spectroscopic profiling variations in extracellular vesicle biochemistry in a model of myogenesis. Journal of Tissue Engineering, 12, 20417314211022092. 10.1177/20417314211022092 34104390 PMC8172953

[jex2171-bib-0022] De Gasperi, R. , *et al.* (2017). Denervation‐related alterations and biological activity of miRNAs contained in exosomes released by skeletal muscle fibers. Scientific Reports, 7, 12888. 29038428 10.1038/s41598-017-13105-9 PMC5643439

[jex2171-bib-0023] DeVito, S. , Woodrick, J. , Song, L. , & Roy, R. (2017). Mutagenic potential of hypoxanthine in live human cells. Mutation Research, 803‐805, 9–16. 10.1016/j.mrfmmm.2017.06.005 PMC5623627 28704682

[jex2171-bib-0024] Distler, U. , Kuharev, J. , Navarro, P. , Levin, Y. , Schild, H. , & Tenzer, S. (2014). Drift time‐specific collision energies enable deep‐coverage data‐independent acquisition proteomics. Nature Methods, 11(2), 167–170. 10.1038/nmeth.2767 24336358

[jex2171-bib-0025] Enciso‐Martinez, A. , Van Der Pol, E. , Hau, C. M. , Nieuwland, R. , Van Leeuwen, T. G. , Terstappen, L. W. M. M. , & Otto, C. (2020). Label‐free identification and chemical characterisation of single extracellular vesicles and lipoproteins by synchronous Rayleigh and Raman scattering. Journal of Extracellular Vesicles, 9(1), 1730134. 10.1080/20013078.2020.1730134 32158522 PMC7048173

[jex2171-bib-0026] Estébanez, B. , Jiménez‐Pavón, D. , Huang, C. J. , Cuevas, M. J. , & González‐Gallego, J. (2021). Effects of exercise on exosome release and cargo in in vivo and ex vivo models: A systematic review. Journal of Cellular Physiology, 236, 3336–3353. 10.1002/jcp.30094 33037627

[jex2171-bib-0027] Ethgen, O. , Beaudart, C. , Buckinx, F. , Bruyère, O. , & Reginster, J. Y. (2017). The future prevalence of sarcopenia in Europe: A claim for public health action. Calcified Tissue International, 100(3), 229–234. 10.1007/s00223-016-0220-9 28012107 PMC5313588

[jex2171-bib-0028] Fei, F. , Zhu, D. L. , Tao, L. J. , Huang, B. Z. , & Zhang, H. H. (2015). Protective effect of ATP on skeletal muscle satellite cells damaged by H₂O₂. Journal of Huazhong University of Science and Technology—Medical Science, 35(1), 76–81. 10.1007/s11596-015-1392-7 25673197

[jex2171-bib-0029] Fernández‐Rhodes, M. , Adlou, B. , Williams, S. , Lees, R. , Peacock, B. , Aubert, D. , Jalal, A. R. , Lewis, M. P. , & Davies, O. G. (2023). Defining the influence of size‐exclusion chromatography fraction window and ultrafiltration column choice on extracellular vesicle recovery in a skeletal muscle model. Journal of Extracellular Biology, 2(4), 1–18. 10.1002/jex2.85 PMC11080914 38939692

[jex2171-bib-0030] Fernández‐Rhodes, M. , Adlou, B. , Williams, S. , Lees, R. , Peacock, B. , Aubert, D. , Jalal, A. R. , Lewis, M. P. , & Davies, O. G. (2023). Defining the influence of size‐exclusion chromatography fraction window and ultrafiltration column choice on extracellular vesicle recovery in a skeletal muscle model. Journal of Extracellular Biology, 2(4), 1–18. 10.1002/jex2.85 PMC11080914 38939692

[jex2171-bib-0031] Fleming, J. W. , Capel, A. J. , Rimington, R. P. , Wheeler, P. , Leonard, A. N. , Bishop, N. C. , Davies, O. G. , & Lewis, M. P. (2020). Bioengineered human skeletal muscle capable of functional regeneration. BMC Biology, 18(1), 145. 10.1186/s12915-020-00884-3 33081771 PMC7576716

[jex2171-bib-0032] Fleming, J. W. , Capel, A. J. , Rimington, R. P. , Wheeler, P. , Leonard, A. N. , Bishop, N. C. , Davies, O. G. , & Lewis, M. P. (2020). Bioengineered human skeletal muscle capable of functional regeneration. BMC Biology, 18(1), 145. 10.1186/s12915-020-00884-3 33081771 PMC7576716

[jex2171-bib-0033] Forterre, A. , Jalabert, A. , Berger, E. , Baudet, M. , Chikh, K. , Errazuriz, E. , De Larichaudy, J. , Chanon, S. , Weiss‐Gayet, M. , Hesse, A. M. , Record, M. , Geloen, A. , Lefai, E. , Vidal, H. , Couté, Y. , & Rome, S. (2014). Proteomic analysis of C2C12 myoblast and myotube exosome‐like vesicles: A new paradigm for myoblast‐myotube cross talk? PLoS ONE, 9(1), e84153. 10.1371/journal.pone.0084153 24392111 PMC3879278

[jex2171-bib-0034] Frühbeis, C. , Helmig, S. , Tug, S. , Simon, P. , & Krämer‐Albers, E. M. (2015). Physical exercise induces rapid release of small extracellular vesicles into the circulation. Journal of Extracellular Vesicles, 4, 1–11. 10.3402/jev.v4.28239 PMC4491306 26142461

[jex2171-bib-0035] Fry, C. S. , Kirby, T. J. , Kosmac, K. , McCarthy, J. J. , & Peterson, C. A. (2017). Myogenic progenitor cells control extracellular matrix production by fibroblasts during skeletal muscle hypertrophy. Cell Stem Cell, 20(1), 56–69. 10.1016/j.stem.2016.09.010 27840022 PMC5218963

[jex2171-bib-0036] Fulzele, S. , Mendhe, B. , Khayrullin, A. , Johnson, M. , Kaiser, H. , Liu, Y. , Isales, C. M. , & Hamrick, M. W. (2019). Muscle‐derived miR‐34a increases with age in circulating extracellular vesicles and induces senescence of bone marrow stem cells. Aging, 11(6), 1791–1803. 10.18632/aging.101874 30910993 PMC6461183

[jex2171-bib-0037] Garner, R. T. , Solfest, J. S. , Nie, Y. , Kuang, S. , Stout, J. , & Gavin, T. P. (2020). Multivesicular body and exosome pathway responses to acute exercise. Experimental Physiology, 105(3), 511–521. 10.1113/EP088017 31917487

[jex2171-bib-0038] Gelibter, S. , Marostica, G. , Mandelli, A. , Siciliani, S. , Podini, P. , Finardi, A. , & Furlan, R. (2022). The impact of storage on extracellular vesicles: A systematic study. Journal of Extracellular Vesicles, 11(2), e12162. 10.1002/jev2.12162 35102719 PMC8804350

[jex2171-bib-0039] Gomarasca, M. , Banfi, G. , & Lombardi, G. (2020). Myokines: The endocrine coupling of skeletal muscle and bone. Advances in Clinical Chemistry, 94, 155–218. 10.1016/bs.acc.2019.07.010 31952571

[jex2171-bib-0040] Görgens, A. , Corso, G. , Hagey, D. W. , Wiklander, J. , Gustafsson, M. O. , Felldin, U. , Lee, Y. , Bostancioglu, R. B. , Sork, H. , Liang, X. , Zheng, W. , Mohammad, D. K. , van de Wakker, S. I. , Vader, P. , Zickler, A. M. , Mamand, D. R. , Ma, L. , Holme, M. N. , Stevens, M. M. , … El Andaloussi, S. (2022). Identification of storage conditions stabilizing extracellular vesicles preparations. Journal of Extracellular Vesicles, 11(6), e12238. 10.1002/jev2.12238 35716060 PMC9206228

[jex2171-bib-0041] Gualerzi, A. , Niada, S. , Giannasi, C. , Picciolini, S. , Morasso, C. , Vanna, R. , Rossella, V. , Masserini, M. , Bedoni, M. , Ciceri, F. , Bernardo, M. E. , Brini, A. T. , & Gramatica, F. (2017). Raman spectroscopy uncovers biochemical tissue‐related features of extracellular vesicles from mesenchymal stromal cells. Scientific Reports, 7(1), 9820. 10.1038/s41598-017-10448-1 28852131 PMC5575260

[jex2171-bib-0042] Guescini, M. , Canonico, B. , Lucertini, F. , Maggio, S. , Annibalini, G. , Barbieri, E. , Luchetti, F. , Papa, S. , & Stocchi, V. (2015). Muscle releases alpha‐sarcoglycan positive extracellular vesicles carrying miRNAs in the bloodstream. PLoS ONE, 10(5), e0125094. 10.1371/journal.pone.0125094 25955720 PMC4425492

[jex2171-bib-0043] Guescini, M. , Maggio, S. , Ceccaroli, P. , Battistelli, M. , Annibalini, G. , Piccoli, G. , Sestili, P. , & Stocchi, V. (2017). Extracellular vesicles released by oxidatively injured or intact C2C12 myotubes promote distinct responses converging toward myogenesis. International Journal of Molecular Sciences, 18(11), 2488. 10.3390/ijms18112488 29165341 PMC5713454

[jex2171-bib-0044] Hanson, B. , Vorobieva, I. , Zheng, W. , Conceição, M. , Lomonosova, Y. , Mäger, I. , Puri, P. L. , El Andaloussi, S. , Wood, M. J. A. , & Roberts, T. C. (2023). EV‐mediated promotion of myogenic differentiation is dependent on dose, collection medium, and isolation method. Molecular Therapy Nucleic Acids, 33, 511–528. 10.1016/j.omtn.2023.07.005 37602275 PMC10432918

[jex2171-bib-0045] Hettinger, Z. R. , Kargl, C. K. , Shannahan, J. H. , Kuang, S. , & Gavin, T. P. (2021). Extracellular vesicles released from stress‐induced prematurely senescent myoblasts impair endothelial function and proliferation. Experimental Physiology, 106(10), 2083–2095. 10.1113/EP089423 34333817

[jex2171-bib-0046] Huang, H. , Ma, S. , Xing, X. , Su, X. , Xu, X. , Tang, Q. , Gao, X. , Yang, J. , Li, M. , Liang, C. , Wu, Y. , Liao, L. , & Tian, W. (2023). Muscle‐derived extracellular vesicles improve disuse‐induced osteoporosis by rebalancing bone formation and bone resorption. Acta Biomaterialia, 157, 609–624. 10.1016/j.actbio.2022.12.019 36526242

[jex2171-bib-0047] Ichimura, T. , Chiu, L. D. , Fujita, K. , Machiyama, H. , Kawata, S. , Watanabe, T. M. , & Fujita, H. (2015). Visualizing the appearance and disappearance of the attractor of differentiation using Raman spectral imaging. Scientific Reports, 5, 1–3. 10.1038/srep11358 PMC5155549 26079396

[jex2171-bib-0048] Jackson, M. J. , Pollock, N. , Staunton, C. , Jones, S. , & McArdle, A. (2022). Redox control of signalling responses to contractile activity and ageing in skeletal muscle. Cells, 11(10), 1698. 35626735 10.3390/cells11101698 PMC9139227

[jex2171-bib-0049] Jalabert, A. , Reininger, L. , Berger, E. , Coute, Y. , Meugnier, E. , Forterre, A. , Errazuriz‐Cerda, E. , Geloen, A. , Aouadi, M. , Bouzakri, K. , Rieusset, J. , & Rome, S. (2021). Profiling of ob/ob mice skeletal muscle exosome‐like vesicles demonstrates combined action of miRNAs, proteins and lipids to modulate lipid homeostasis in recipient cells. Scientific Reports, 11(1), 21626. 10.1038/s41598-021-00983-3 34732797 PMC8566600

[jex2171-bib-0050] Jung, J. H. , Lee, M. Y. , Choi, D. Y. , Lee, J. W. , You, S. , Lee, K. Y. , Kim, J. , & Kim, K. P. (2015). Phospholipids of tumor extracellular vesicles stratify gefitinib‐resistant nonsmall cell lung cancer cells from gefitinib‐sensitive cells. Proteomics, 15(4), 824–835. 10.1002/pmic.201400243 25404199 PMC5305024

[jex2171-bib-0051] Karvinen, S. , Korhonen, T. M. , Sievänen, T. , Karppinen, J. E. , Juppi, H. K. , Jakoaho, V. , Kujala, U. M. , Laukkanen, J. A. , Lehti, M. , & Laakkonen, E. K. (2023). Extracellular vesicles and high‐density lipoproteins: Exercise and oestrogen‐responsive small RNA carriers. Journal of Extracellular Vesicles, 12(2), e12308. 10.1002/jev2.12308 36739598 PMC9899444

[jex2171-bib-0052] Kinoshita, H. , Orita, S. , Inage, K. , Yamauchi, K. , Abe, K. , Inoue, M. , Norimoto, M. , Umimura, T. , Eguchi, Y. , Fujimoto, K. , Shiga, Y. , Kanamoto, H. , Aoki, Y. , Furuya, T. , Suzuki, M. , Akazawa, T. , Takahashi, K. , & Ohtori, S. (2019). Skeletal muscle cell oxidative stress as a possible therapeutic target in a denervation‐induced experimental sarcopenic model. Spine, 44(8), E446–E455. 10.1097/BRS.0000000000002891 30299418

[jex2171-bib-0053] Koster, H. J. , Rojalin, T. , Powell, A. , Pham, D. , Mizenko, R. R. , Birkeland, A. C. , & Carney, R. P. (2021). Surface enhanced Raman scattering of extracellular vesicles for cancer diagnostics despite isolation dependent lipoprotein contamination. Nanoscale, 13(35), 14760–14776. 10.1039/d1nr03334d 34473170 PMC8447870

[jex2171-bib-0054] Kowal, J. , Arras, G. , Colombo, M. , Jouve, M. , Morath, J. P. , Primdal‐Bengtson, B. , Dingli, F. , Loew, D. , Tkach, M. , & Théry, C. (2016). Proteomic comparison defines novel markers to characterize heterogeneous populations of extracellular vesicle subtypes. Proceedings of the National Academy of Sciences of the United States of America, 113(8), E968–E977. 10.1073/pnas.1521230113 26858453 PMC4776515

[jex2171-bib-0055] Lamichhane, T. N. , Sokic, S. , Schardt, J. S. , Raiker, R. S. , Lin, J. W. , & Jay, S. M. (2015). Emerging roles for extracellular vesicles in tissue engineering and regenerative medicine. Tissue Engineering. Part B, Reviews, 21(1), 45–54. 10.1089/ten.TEB.2014.0300 24957510 PMC4321981

[jex2171-bib-0056] Lara‐Castillo, N. , & Johnson, M. L. (2020). Bone‐muscle mutual interactions. Current Osteoporosis Reports, 18(4), 408–421. 10.1007/s11914-020-00602-6 32519283 PMC8059358

[jex2171-bib-0057] Larsson, L. , Degens, H. , Li, M. , Salviati, L. , Lee, Y. I. , Thompson, W. , Kirkland, J. L. , & Sandri, M. (2019). Sarcopenia: Aging‐related loss of muscle mass and function. Physiological Reviews, 99(1), 427–511. 10.1152/physrev.00061.2017 30427277 PMC6442923

[jex2171-bib-0058] Le Bihan, M. C. , Bigot, A. , Jensen, S. S. , Dennis, J. L. , Rogowska‐Wrzesinska, A. , Lainé, J. , Gache, V. , Furling, D. , Jensen, O. N. , Voit, T. , Mouly, V. , Coulton, G. R. , & Butler‐Browne, G. (2012). In‐depth analysis of the secretome identifies three major independent secretory pathways in differentiating human myoblasts. Journal of Proteomics, 77, 344–356. 10.1016/j.jprot.2012.09.008 23000592

[jex2171-bib-0059] Lee, H. , Kim, S. H. , Lee, J. S. , Yang, Y. H. , Nam, J. M. , Kim, B. W. , & Ko, Y. G. (2016). Mitochondrial oxidative phosphorylation complexes exist in the sarcolemma of skeletal muscle. BMB Reports, 49(2), 116–121. 10.5483/bmbrep.2016.49.2.232 26645635 PMC4915115

[jex2171-bib-0060] Lee, S. M. , Lee, S. H. , Jung, Y. , Lee, Y. , Yoon, J. H. , Choi, J. Y. , Hwang, C. Y. , Son, Y. H. , Park, S. S. , Hwang, G. S. , Lee, K. P. , & Kwon, K. S. (2020). FABP3‐mediated membrane lipid saturation alters fluidity and induces ER stress in skeletal muscle with aging. Nature Communications, 11(1), 1–15. 10.1038/s41467-020-19501-6 PMC7653047 33168829

[jex2171-bib-0061] Lees, R. , Tempest, R. , Law, A. , Aubert, D. , Davies, O. G. , Williams, S. , Peake, N. , & Peacock, B. (2022). Single extracellular vesicle transmembrane protein characterization by nano‐flow cytometry. Journal of Visualized Experiments: JoVE, (185), e64020. 10.3791/64020 35969098

[jex2171-bib-0062] Legoedec, J. , Gasque, P. , Jeanne, J. F. , Scotte, M. , & Fontaine, M. (1997). Complement classical pathway expression by human skeletal myoblasts in vitro. Molecular Immunology, 34(10), 735–741. 10.1016/s0161-5890(97)00093-x 9430201

[jex2171-bib-0063] Li, G. Z. , Vissers, J. P. , Silva, J. C. , Golick, D. , Gorenstein, M. V. , & Geromanos, S. J. (2009). Database searching and accounting of multiplexed precursor and product ion spectra from the data independent analysis of simple and complex peptide mixtures. Proteomics, 9(6), 1696–1719. 10.1002/pmic.200800564 19294629

[jex2171-bib-0064] Lian, D. , Chen, M. M. , Wu, H. , Deng, S. , & Hu, X. (2022). The role of oxidative stress in skeletal muscle myogenesis and muscle disease. Antioxidants, 11, 755. 10.3390/antiox11040755 35453440 PMC9026549

[jex2171-bib-0065] Lischnig, A. , Bergqvist, M. , Ochiya, T. , & Lässer, C. (2022). Quantitative proteomics identifies proteins enriched in large and small extracellular vesicles. Molecular & Cellular Proteomics: MCP, 21(9), 100273. 10.1016/j.mcpro.2022.100273 35918030 PMC9486130

[jex2171-bib-0066] Liu, L. , Kawashima, M. , Sugimoto, M. , Sonomura, K. , Pu, F. , Li, W. , Takeda, M. , Goto, T. , Kawaguchi, K. , Sato, T. A. , & Toi, M. (2023). Discovery of lipid profiles in plasma‐derived extracellular vesicles as biomarkers for breast cancer diagnosis. Cancer Science, 114(10), 4020–4031. 10.1111/cas.15935 37608343 PMC10551607

[jex2171-bib-0067] Ludwig, N. , Gillespie, D. G. , Reichert, T. E. , Jackson, E. K. , & Whiteside, T. L. (2020). Purine metabolites in tumor‐derived exosomes may facilitate immune escape of head and neck squamous cell carcinoma. Cancers (Basel), 12, 1–14. 10.3390/cancers12061602 PMC7352909 32560461

[jex2171-bib-0068] Maggio, S. , Canonico, B. , Ceccaroli, P. , Polidori, E. , Cioccoloni, A. , Giacomelli, L. , Ferri Marini, C. , Annibalini, G. , Gervasi, M. , Benelli, P. , Fabbri, F. , Del Coco, L. , Fanizzi, F. P. , Giudetti, A. M. , Lucertini, F. , & Guescini, M. (2023). Modulation of the circulating extracellular vesicles in response to different exercise regimens and study of their inflammatory effects. International Journal of Molecular Sciences, 24(3), 3039. 10.3390/ijms24033039 36769362 PMC9917742

[jex2171-bib-0069] Maroto, R. , Zhao, Y. , Jamaluddin, M. , Popov, V. L. , Wang, H. , Kalubowilage, M. , Zhang, Y. , Luisi, J. , Sun, H. , Culbertson, C. T. , Bossmann, S. H. , Motamedi, M. , & Brasier, A. R. (2017). Effects of storage temperature on airway exosome integrity for diagnostic and functional analyses. Journal of Extracellular Vesicles, 6(1), 1359478. 10.1080/20013078.2017.1359478 28819550 PMC5556670

[jex2171-bib-0070] Martin, N. R. , Passey, S. L. , Player, D. J. , Khodabukus, A. , Ferguson, R. A. , Sharples, A. P. , Mudera, V. , Baar, K. , & Lewis, M. P. (2013). Factors affecting the structure and maturation of human tissue engineered skeletal muscle. Biomaterials, 34(23), 5759–5765. 10.1016/j.biomaterials.2013.04.002 23643182

[jex2171-bib-0071] Matsuzaka, Y. , Tanihata, J. , Komaki, H. , Ishiyama, A. , Oya, Y. , Rüegg, U. , Takeda, S. I. , & Hashido, K. (2016). Characterization and functional analysis of extracellular vesicles and muscle‐abundant miRNAs (miR‐1, miR‐133a, and miR‐206) in C2C12 myocytes and mdx mice. PLoS ONE, 11(12), e0167811. 10.1371/journal.pone.0167811 27977725 PMC5158003

[jex2171-bib-0072] Maurel, D. B. , Jähn, K. , & Lara‐Castillo, N. (2017). Muscle‐bone crosstalk: Emerging opportunities for novel therapeutic approaches to treat musculoskeletal pathologies. Biomedicines, 5(4), 62. 10.3390/biomedicines5040062 29064421 PMC5744086

[jex2171-bib-0073] McArdle, A. , & Jackson, M. J. (2019). An introduction to a special issue of free radical biology and medicine—“reactive oxygen species and musculoskeletal aging”. Free radical Biology & Medicine, 132, 1–2. 30665616 10.1016/j.freeradbiomed.2018.12.038

[jex2171-bib-0074] Midekessa, G. , Godakumara, K. , Ord, J. , Viil, J. , Lättekivi, F. , Dissanayake, K. , Kopanchuk, S. , Rinken, A. , Andronowska, A. , Bhattacharjee, S. , Rinken, T. , & Fazeli, A. (2020). Zeta potential of extracellular vesicles: Toward understanding the attributes that determine colloidal stability. ACS Omega, 5(27), 16701–16710. 32685837 10.1021/acsomega.0c01582 PMC7364712

[jex2171-bib-0075] Mizenko, R. R. , Brostoff, T. , Rojalin, T. , Koster, H. J. , Swindell, H. S. , Leiserowitz, G. S. , Wang, A. , & Carney, R. P. (2021). Tetraspanins are unevenly distributed across single extracellular vesicles and bias sensitivity to multiplexed cancer biomarkers. Journal of Nanobiotechnology, 19(1), 250. 10.1186/s12951-021-00987-1 34419056 PMC8379740

[jex2171-bib-0076] Muratore, M. , Srsen, V. , Waterfall, M. , Downes, A. , & Pethig, R. (2012). Biomarker‐free dielectrophoretic sorting of differentiating myoblast multipotent progenitor cells and their membrane analysis by Raman spectroscopy. Biomicrofluidics, 6, 1–16. 10.1063/1.4746252 PMC3432085 23940503

[jex2171-bib-0077] Murphy, D. P. , Nicholson, T. , Jones, S. W. , & O'Leary, M. F. (2019). MyoCount: A software tool for the automated quantification of myotube surface area and nuclear fusion index. Wellcome Open Research, 4, 6. 10.12688/wellcomeopenres.15055.1 30906880 PMC6419977

[jex2171-bib-0078] Mytidou, C. , Koutsoulidou, A. , Katsioloudi, A. , Prokopi, M. , Kapnisis, K. , Michailidou, K. , Anayiotos, A. , & Phylactou, L. A. (2021). Muscle‐derived exosomes encapsulate myomiRs and are involved in local skeletal muscle tissue communication. FASEB Journal: Official Publication of the Federation of American Societies for Experimental Biology, 35(2), e21279. 10.1096/fj.201902468RR 33484211 PMC12315491

[jex2171-bib-0079] Mytidou, C. , Koutsoulidou, A. , Zachariou, M. , Prokopi, M. , Kapnisis, K. , Spyrou, G. M. , Anayiotos, A. , & Phylactou, L. A. (2021). Age‐related exosomal and endogenous expression patterns of miR‐1, miR‐133a, miR‐133b, and miR‐206 in skeletal muscles. Frontiers in Physiology, 12, 1–12. 10.3389/fphys.2021.708278 PMC8637414 34867435

[jex2171-bib-0080] Olshansky, S. J. , Goldman, D. P. , Zheng, Y. , & Rowe, J. W. (2008). Aging in America in the twenty‐first century: Demographic forecasts from the MacArthur Foundation Research Network on an Aging Society. The Milbank Quarterly, 86, 529–532. 20021588 10.1111/j.1468-0009.2009.00581.x PMC2888016

[jex2171-bib-0081] Parlatan, U. , Ozen, M. O. , Kecoglu, I. , Koyuncu, B. , Torun, H. , Khalafkhany, D. , Loc, I. , Ogut, M. G. , Inci, F. , Akin, D. , Solaroglu, I. , Ozoren, N. , Unlu, M. B. , & Demirci, U. (2023). Label‐free identification of exosomes using raman spectroscopy and machine learning. Small (Weinheim an Der Bergstrasse, Germany), 19(9), 1–12. 10.1002/smll.202205519 36642804

[jex2171-bib-0082] Raposo, G. , & Stahl, P. D. (2019). Extracellular vesicles: A new communication paradigm? Nature Reviews. Molecular Cell Biology, 20(9), 509–510. 10.1038/s41580-019-0158-7 31324871

[jex2171-bib-0083] Raposo, G. , & Stoorvogel, W. (2013). Extracellular vesicles: Exosomes, microvesicles, and friends. The Journal of Cell Biology, 200(4), 373–383. 10.1083/jcb.201211138 23420871 PMC3575529

[jex2171-bib-0084] Rimington, R. P. , Fleming, J. W. , Capel, A. J. , Wheeler, P. C. , & Lewis, M. P. (2021). Bioengineered model of the human motor unit with physiologically functional neuromuscular junctions. Scientific Reports, 11(1), 11695. 34083648 10.1038/s41598-021-91203-5 PMC8175425

[jex2171-bib-0085] Rimington, R. P. , Fleming, J. W. , Capel, A. J. , Wheeler, P. C. , & Lewis, M. P. (2021). Bioengineered model of the human motor unit with physiologically functional neuromuscular junctions. Scientific Reports, 11(1), 11695. 10.1038/s41598-021-91203-5 34083648 PMC8175425

[jex2171-bib-0086] Rodriguez‐Suarez, E. , Hughes, C. , Gethings, L. , Giles, K. , Wildgoose, J. , Stapels, M. , Fadgen, K. E. , Geromanos, S. J. , Vissers, J. P. C. , Elortza, F. , & Langridge, J. I. (2013). An ion mobility assisted data independent LC‐MS strategy for the analysis of complex biological samples. Current Analytical Chemistry, 9, 199–211.

[jex2171-bib-0087] Rome, S. (2022). Muscle and adipose tissue communicate with extracellular vesicles. International Journal of Molecular Sciences, 23(13), 7052. 10.3390/ijms23137052 35806052 PMC9266961

[jex2171-bib-0088] Rome, S. , Forterre, A. , Mizgier, M. L. , & Bouzakri, K. (2019). Skeletal muscle‐released extracellular vesicles: State of the art. Frontiers in Physiology, 10, 929. 10.3389/fphys.2019.00929 31447684 PMC6695556

[jex2171-bib-0089] Safdar, A. , & Tarnopolsky, M. A. (2018). Exosomes as mediators of the systemic adaptations to endurance exercise. Cold Spring Harbor Perspectives in Medicine, 8(3), 1–23. 10.1101/cshperspect.a029827 PMC5830902 28490541

[jex2171-bib-0090] Sahu, A. , Clemens, Z. J. , Shinde, S. N. , Sivakumar, S. , Pius, A. , Bhatia, A. , Picciolini, S. , Carlomagno, C. , Gualerzi, A. , Bedoni, M. , Van Houten, B. , Lovalekar, M. , Fitz, N. F. , Lefterov, I. , Barchowsky, A. , Koldamova, R. , & Ambrosio, F. (2021). Regulation of aged skeletal muscle regeneration by circulating extracellular vesicles. Nature Aging, 1(12), 1148–1161. 10.1038/s43587-021-00143-2 35665306 PMC9165723

[jex2171-bib-0091] Sen, A. , Youssef, S. , Wendt, K. , & Anakk, S. (2023). Depletion of IQ motif‐containing GTPase activating protein 2 (IQGAP2) reduces hepatic glycogen and impairs insulin signaling. Journal of Biological Chemistry, 299(11), 105322. 10.1016/j.jbc.2023.105322 37805137 PMC10652104

[jex2171-bib-0092] Sergeeva, O. , & Zatsepin, T. (2021). Rna helicases as shadow modulators of cell cycle progression. International Journal of Molecular Sciences, 22, 1–16. 10.3390/ijms22062984 PMC8001981 33804185

[jex2171-bib-0093] Severinsen, M. C. K. , & Pedersen, B. K. (2020). Muscle‐Organ Crosstalk: The emerging roles of myokines. Endocrine Reviews, 41(4), 594–609. 10.1210/endrev/bnaa016 32393961 PMC7288608

[jex2171-bib-0094] Shaba, E. , Vantaggiato, L. , Governini, L. , Haxhiu, A. , Sebastiani, G. , Fignani, D. , Grieco, G. E. , Bergantini, L. , Bini, L. , & Landi, C. (2022). Multi‐omics integrative approach of extracellular vesicles: A future challenging milestone. Proteomes, 10(2), 12. 10.3390/proteomes10020012 35645370 PMC9149947

[jex2171-bib-0095] Shao, X. , Gong, W. , Wang, Q. , Wang, P. , Shi, T. , Mahmut, A. , Qin, J. , Yao, Y. , Yan, W. , Chen, D. , Chen, X. , Jiang, Q. , & Guo, B. (2022). Atrophic skeletal muscle fibre‐derived small extracellular vesicle miR‐690 inhibits satellite cell differentiation during ageing. Journal of Cachexia, Sarcopenia and Muscle, 13(6), 3163–3180. 10.1002/jcsm.13106 36237168 PMC9745557

[jex2171-bib-0096] Sharples, A. P. , Player, D. J. , Martin, N. R. , Mudera, V. , Stewart, C. E. , & Lewis, M. P. (2012). Modelling in vivo skeletal muscle ageing in vitro using three‐dimensional bioengineered constructs. Aging Cell, 11(6), 986–995. 10.1111/j.1474-9726.2012.00869.x 22882433

[jex2171-bib-0097] Sies, H. , & Jones, D. P. (2020). Reactive oxygen species (ROS) as pleiotropic physiological signalling agents. Nature Reviews. Molecular Cell Biology, 21(7), 363–383. 10.1038/s41580-020-0230-3 32231263

[jex2171-bib-0098] Sinanan, A. C. , Hunt, N. P. , & Lewis, M. P. (2004). Human adult craniofacial muscle‐derived cells: Neural‐cell adhesion‐molecule (NCAM; CD56)‐expressing cells appear to contain multipotential stem cells. Biotechnology and Applied Biochemistry, 40(Pt 1), 25–34. 10.1042/BA20030185 15270704

[jex2171-bib-0099] Skovronova, R. , Grange, C. , Dimuccio, V. , Deregibus, M. C. , Camussi, G. , & Bussolati, B. (2021). Surface marker expression in small and medium/large mesenchymal stromal cell‐derived extracellular vesicles in naive or apoptotic condition using orthogonal techniques. Cells, 10(11), 2948. 10.3390/cells10112948 34831170 PMC8616318

[jex2171-bib-0100] Smith, J. M. , Hedman, A. C. , & Sacks, D. B. (2015). IQGAPs choreograph cellular signaling from the membrane to the nucleus. Trends in Cell Biology, 25, 171–184. 25618329 10.1016/j.tcb.2014.12.005 PMC4344846

[jex2171-bib-0101] Sparks, D. L. , Chatterjee, C. , Young, E. , Renwick, J. , & Pandey, N. R. (2008). Lipoprotein charge and vascular lipid metabolism. Chemistry and Physics of Lipids, 154(1), 1–6. 10.1016/j.chemphyslip.2008.04.006 18502203

[jex2171-bib-0102] Su, H. , Masters, C. L. , Bush, A. I. , Barnham, K. J. , Reid, G. E. , & Vella, L. J. (2024). Exploring the significance of lipids in Alzheimer's disease and the potential of extracellular vesicles. Proteomics, 24(11), 2300063. 10.1002/pmic.202300063 37654087

[jex2171-bib-0103] Tadokoro, H. , Hirayama, A. , Kudo, R. , Hasebe, M. , Yoshioka, Y. , Matsuzaki, J. , Yamamoto, Y. , Sugimoto, M. , Soga, T. , & Ochiya, T. (2020). Adenosine leakage from perforin‐burst extracellular vesicles inhibits perforin secretion by cytotoxic T‐lymphocytes. PLoS ONE, 15(4), e0231430. 10.1371/journal.pone.0231430 32275689 PMC7147783

[jex2171-bib-0104] Takagi, H. , Ikehara, T. , Hashimoto, K. , Tanimoto, K. , Shimazaki, A. , Kashiwagi, Y. , Sakamoto, S. , & Yukioka, H. (2021). Acetyl‐CoA carboxylase 2 inhibition reduces skeletal muscle bioactive lipid content and attenuates progression of type 2 diabetes in Zucker diabetic fatty rats. European Journal of Pharmacology, 910, 174451. 10.1016/j.ejphar.2021.174451 34454928

[jex2171-bib-0105] Tarnopolsky, M. A. , Pearce, E. , Smith, K. , & Lach, B. (2011). Suction‐modified Bergström muscle biopsy technique: Experience with 13,500 procedures. Muscle & Nerve, 43(5), 717–725. 10.1002/mus.21945 21462204

[jex2171-bib-0106] Taylor, D. D. , & Shah, S. (2015). Methods of isolating extracellular vesicles impact down‐stream analyses of their cargoes. Methods (San Diego, Calif.), 87, 3–10. 10.1016/j.ymeth.2015.02.019 25766927

[jex2171-bib-0107] Théry, C. , Witwer, K. W. , Aikawa, E. , Alcaraz, M. J. , Anderson, J. D. , Andriantsitohaina, R. , Antoniou, A. , Arab, T. , Archer, F. , Atkin‐Smith, G. K. , Ayre, D. C. , Bach, J. M. , Bachurski, D. , Baharvand, H. , Balaj, L. , Baldacchino, S. , Bauer, N. N. , Baxter, A. A. , Bebawy, M. , … Zuba‐Surma, E. K. (2018). Minimal information for studies of extracellular vesicles 2018 (MISEV2018): A position statement of the International Society for Extracellular Vesicles and update of the MISEV2014 guidelines. Journal of Extracellular Vesicles, 7(1), 1535750. 10.1080/20013078.2018.1535750 30637094 PMC6322352

[jex2171-bib-0108] Trovato, E. , Di Felice, V. , & Barone, R. (2019). Extracellular vesicles: Delivery vehicles of myokines. Frontiers in Physiology, 10, 522. 10.3389/fphys.2019.00522 31133872 PMC6514434

[jex2171-bib-0109] Uchitomi, R. , Hatazawa, Y. , Senoo, N. , Yoshioka, K. , Fujita, M. , Shimizu, T. , Miura, S. , Ono, Y. , & Kamei, Y. (2019). Metabolomic analysis of skeletal muscle in aged mice. Scientific Reports, 9(1), 1–11. 10.1038/s41598-019-46929-8 31320689 PMC6639307

[jex2171-bib-0110] Varshosaz, Z. , Abdi, S. , Moazen, E. , & Razavi, A. E. (2012). Human serum lipoprotein zeta potential measurement by zetasizer instrument, a method development. Research in Pharmaceutical Sciences, 7, 626.

[jex2171-bib-0111] Vechetti, I. J. Jr. , Wen, Y. , Chaillou, T. , Murach, K. A. , Alimov, A. P. , Figueiredo, V. C. , Dal‐Pai‐Silva, M. , & McCarthy, J. J. (2019). Life‐long reduction in myomiR expression does not adversely affect skeletal muscle morphology. Scientific Reports, 9(1), 1–11. 10.1038/s41598-019-41476-8 30940834 PMC6445125

[jex2171-bib-0112] Vickers, K. C. , Palmisano, B. T. , Shoucri, B. M. , Shamburek, R. D. , & Remaley, A. T. (2011). MicroRNAs are transported in plasma and delivered to recipient cells by high‐density lipoproteins. Nature Cell Biology, 13(4), 423–433. 10.1038/ncb2210 21423178 PMC3074610

[jex2171-bib-0113] Vidal, M. (2019). Exosomes: Revisiting their role as “garbage bags”. Traffic (Copenhagen, Denmark), 20, 815–828. 31418976 10.1111/tra.12687

[jex2171-bib-0114] Wang, W. , Li, M. , Chen, Z. , Xu, L. , Chang, M. , Wang, K. , Deng, C. , Gu, Y. , Zhou, S. , Shen, Y. , Tao, F. , & Sun, H. (2022). Biogenesis and function of extracellular vesicles in pathophysiological processes of skeletal muscle atrophy. Biochemical Pharmacology, 198, 114954. 10.1016/j.bcp.2022.114954 35167807

[jex2171-bib-0115] Welsh, J. A. , Goberdhan, D. C. I. , O'Driscoll, L. , Buzas, E. I. , Blenkiron, C. , Bussolati, B. , Cai, H. , Di Vizio, D. , Driedonks, T. A. P. , Erdbrügger, U. , Falcon‐Perez, J. M. , Fu, Q. L. , Hill, A. F. , Lenassi, M. , Lim, S. K. , Mahoney, M. G. , Mohanty, S. , Möller, A. , Nieuwland, R. , … Witwer, K. W. (2024). Minimal information for studies of extracellular vesicles (MISEV2023): From basic to advanced approaches. Journal of Extracellular Vesicles, 13(2), e12404. 10.1002/jev2.12404 38326288 PMC10850029

[jex2171-bib-0116] Wilson, D. R. , & Green, J. J. (2017). Nanoparticle tracking analysis for determination of hydrodynamic diameter, concentration, and zeta‐potential of polyplex nanoparticles. Methods in Molecular Biology (Clifton, N.J.), 1570, 31–46. 10.1007/978-1-4939-6840-4_3 28238128

[jex2171-bib-0117] Witwer, K. W. , Buzás, E. I. , Bemis, L. T. , Bora, A. , Lässer, C. , Lötvall, J. , Nolte‐’t Hoen, E. N. , Piper, M. G. , Sivaraman, S. , Skog, J. , Théry, C. , Wauben, M. H. , & Hochberg, F. (2013). Standardization of sample collection, isolation and analysis methods in extracellular vesicle research. Journal of Extracellular Vesicles, 2, 20360. 10.3402/jev.v2i0.20360 PMC3760646 24009894

[jex2171-bib-0118] Xhuti, D. , Nilsson, M. I. , Manta, K. , Tarnopolsky, M. A. , & Nederveen, J. P. (2023). Circulating exosome‐like vesicle and skeletal muscle microRNAs are altered with age and resistance training. The Journal of Physiology, 601(22), 5051–5073. 10.1113/JP282663 36722691

[jex2171-bib-0119] Yuan, F. , Li, Y. M. , & Wang, Z. (2021). Preserving extracellular vesicles for biomedical applications: Consideration of storage stability before and after isolation. Drug Delivery, 28(1), 1501–1509. 10.1080/10717544.2021.1951896 34259095 PMC8281093

[jex2171-bib-0120] Yuan, S. , & Larsson, S. C. (2023). Epidemiology of sarcopenia: Prevalence, risk factors, and consequences. Metabolism, 144, 155533. 36907247 10.1016/j.metabol.2023.155533

[jex2171-bib-0121] Yuana, Y. , Levels, J. , Grootemaat, A. , Sturk, A. , & Nieuwland, R. (2014). Co‐isolation of extracellular vesicles and high‐density lipoproteins using density gradient ultracentrifugation. Journal of Extracellular Vesicles, 3, 10.3402/jev.v3.23262 PMC4090368 25018865

[jex2171-bib-0122] Zhang, H. , Freitas, D. , Kim, H. S. , Fabijanic, K. , Li, Z. , Chen, H. , Mark, M. T. , Molina, H. , Martin, A. B. , Bojmar, L. , Fang, J. , Rampersaud, S. , Hoshino, A. , Matei, I. , Kenific, C. M. , Nakajima, M. , Mutvei, A. P. , Sansone, P. , Buehring, W. , … Lyden, D. (2018). Identification of distinct nanoparticles and subsets of extracellular vesicles by asymmetric flow field‐flow fractionation. Nature Cell Biology, 20(3), 332–343. 10.1038/s41556-018-0040-4 29459780 PMC5931706

[jex2171-bib-0123] Zini, J. , Saari, H. , Ciana, P. , Viitala, T. , Lõhmus, A. , Saarinen, J. , & Yliperttula, M. (2022). Infrared and Raman spectroscopy for purity assessment of extracellular vesicles. European Journal of Pharmaceutical Sciences: Official Journal of the European Federation for Pharmaceutical Sciences, 172, 106135. 10.1016/j.ejps.2022.106135 35121019

