# Supplementary material for: Extracellular vesicles may provide an alternative detoxification pathway during skeletal muscle myoblast ageing
Source: J Extracell Biol. 2024 Aug 21;3(8):e171. doi: 10.1002/jex2.171 (PMC11336379; doi:10.1002/jex2.171)
Supplement: Supplementary file 1 — Supporting Information [file JEX2-3-e171-s001.docx]

# **SUPPLEMENTARY FIGURES**


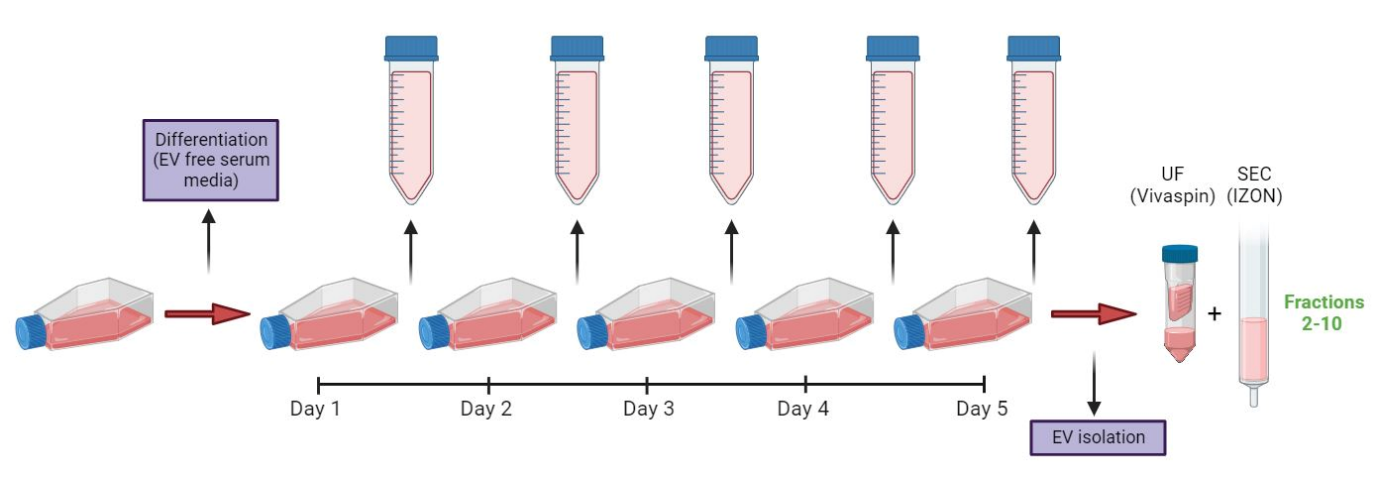


**Supplementary Figure 1:** SM-EV isolation method timeline and protocol.


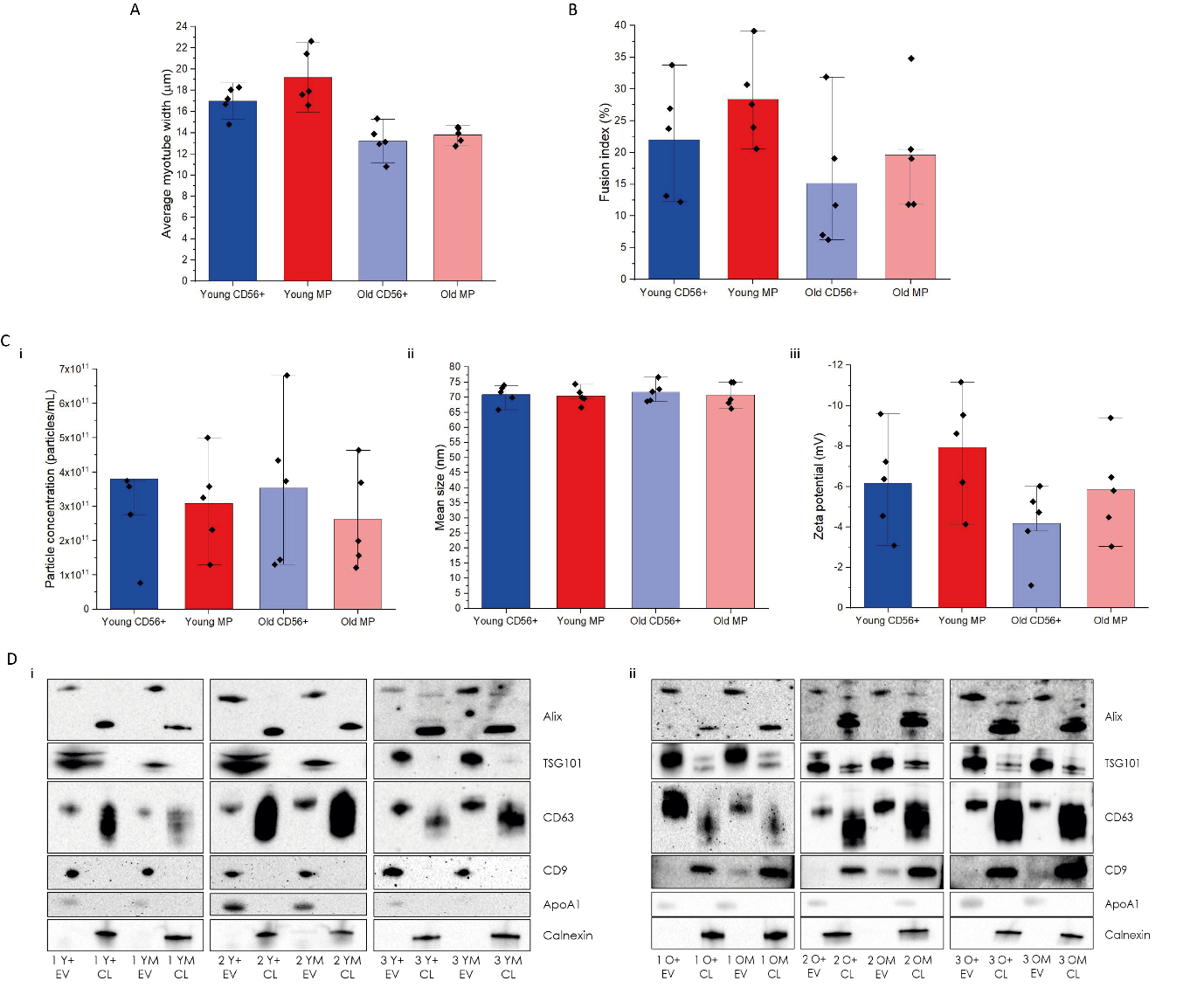


**Supplementary Figure 2: CD56^+^ vs MP comparisons for both age cohorts** (A) Average myotube width. (B) Fusion indices (C) Particle information including (i) Particle concentration, (ii) Mean average size, (iii) Zeta potential. (D) WB of EV markers in SM-EVs and cell lysates (CL) for (i) N=3 young participants and (ii) N=3 old participants.


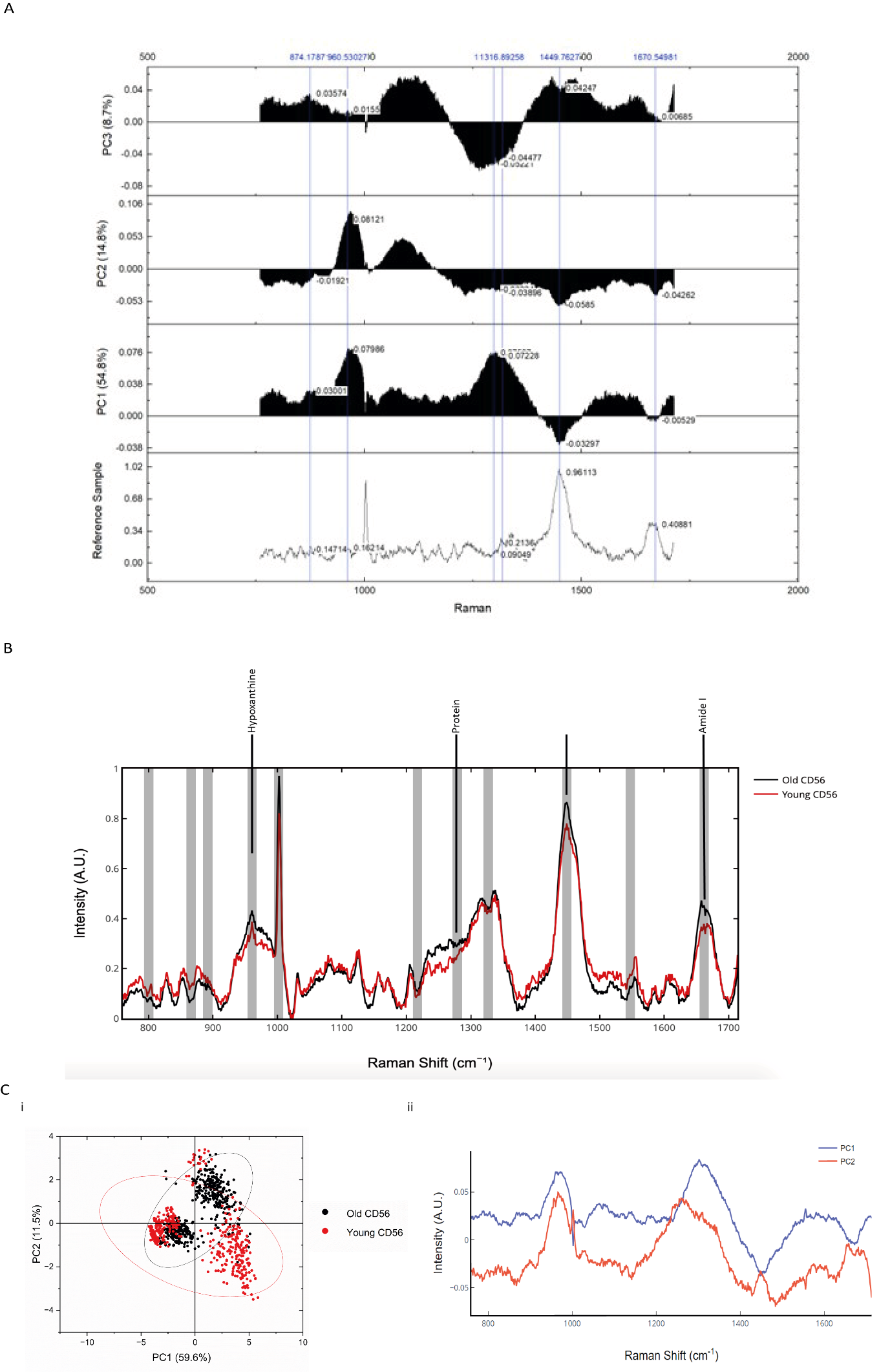


**Supplementary Figure 3: Raman spectroscopy complementary interpretation.** (A) PCA additional information for young and old SM-EVs. (B) Raman spectra (800 to 1700 nm) representing CD56^+^ young and old SM-EVs and main Raman bands illustrated with grey longitudinal bands in Raman and their identifications**.** (B) PCA 2D spatial representation (C) Loadings for 2 PCs. CD56^+^ SM-EVs only had 3 main peaks, 960, 1270 and 1656 cm^-1^.

**Supplementary Table 1: Western Blot antibody guide for targeted proteins.** We included information about dilutions, source, supplier and codes and secondary antibody dilutions used in the results represented in this study.

| Primary Antibody | Source | Dilution | Supplier | Secondary antibody | Dilution |
| --- | --- | --- | --- | --- | --- |
| Anti-Alix | Rabbit | 1:1000 | Santa Cruz (sc-53540) | Anti-Rabbit | 1:3000 |
| Anti- TSG101 | Rabbit | 1:1000 | Abcam (ab30871) | Anti-Rabbit | 1:3000 |
| Anti-CD9 | Rabbit | 1:1000 | Abcam (ab92726) | Anti-Rabbit | 1:2000 |
| Anti-CD63 | Rabbit | 1:1000 | Abcam (ab216130) | Anti-Rabbit | 1:3000 |
| Anti-Calnexin | Mouse | 1:1000 | Abcam (ab22595) | Anti-Mouse | 1:3000 |
| Anti-ApoA1 | Rabbit | 1:1000 | Abcam  (ab20453) | Anti-Rabbit | 1:3000 |

**Supplementary Table 2:** R^2^ values for individual linear regressions

|  |  | Young mixes | | | | |
| --- | --- | --- | --- | --- | --- | --- |
|  | Participant code | 1 | 2 | 3 | 4 | 5 |
| Old mixes | 1 | 0.9652 | 0.9515 | 0.8387 | 0.9881 | 0.9883 |
|  | 2 | 0.9732 | 0.9644 | 0.8526 | 0.9934 | 0.9898 |
|  | 3 | 0.9736 | 0.9559 | 0.8558 | 0.9723 | 0.9845 |
|  | 4 | 0.9705 | 0.963 | 0.9674 | 0.9866 | 0.9928 |
|  | 5 | 0.9732 | 0.9617 | 0.8661 | 0.9842 | 0.9938 |
